# The Influenza A Virus Replication Cycle: A Comprehensive Review

**DOI:** 10.3390/v16020316

**Published:** 2024-02-19

**Authors:** Toby Carter, Munir Iqbal

**Affiliations:** The Pirbright Institute, Ash Road, Pirbright, Woking GU24 0NF, UK; munir.iqbal@pirbright.ac.uk

**Keywords:** influenza A virus, viral replication, viral entry

## Abstract

Influenza A virus (IAV) is the primary causative agent of influenza, colloquially called the flu. Each year, it infects up to a billion people, resulting in hundreds of thousands of human deaths, and causes devastating avian outbreaks with worldwide losses worth billions of dollars. Always present is the possibility that a highly pathogenic novel subtype capable of direct human-to-human transmission will spill over into humans, causing a pandemic as devastating if not more so than the 1918 influenza pandemic. While antiviral drugs for influenza do exist, they target very few aspects of IAV replication and risk becoming obsolete due to antiviral resistance. Antivirals targeting other areas of IAV replication are needed to overcome this resistance and combat the yearly epidemics, which exact a serious toll worldwide. This review aims to summarise the key steps in the IAV replication cycle, along with highlighting areas of research that need more focus.

## 1. Introduction

Influenza A virus (IAV), the major aetiological agent for influenza, is the sole member of the *Alphainfluenzavirus* genus, part of the *Orthomyxoviridae* family of segmented, negative-sense, single-stranded RNA viruses [1]. In humans, it primarily affects the upper respiratory tract and is spread by airborne respiratory droplets, causing hundreds of thousands of deaths annually [2]. The main natural reservoir of IAV is wild aquatic birds, which carry the virus with few, if any, symptoms [3]. In non-reservoir avian species such as poultry, the disease is mostly intestinal and is spread via the faecal–oral route [4]. IAV subtype names refer to which type of haemagglutinin (HA) and neuraminidase (NA), two of the three viral surface proteins, are present in the viral genome. So far, 18 HA and 11 NA subtypes have been described: H1-16 and N1-9 naturally reside in aquatic birds [3]; H17N10 and H18N11 naturally reside in bats [5,6]. Currently, only H1N1 and H3N2 are endemic in humans, but a genetic reassortment might yield a novel subtype, which is both highly pathogenic and capable of direct human-to-human transmission [7]. Such an event has happened five times in the past century, including the 1918 influenza pandemic, which caused an estimated 20 million deaths [8,9].

**Figure 1:**
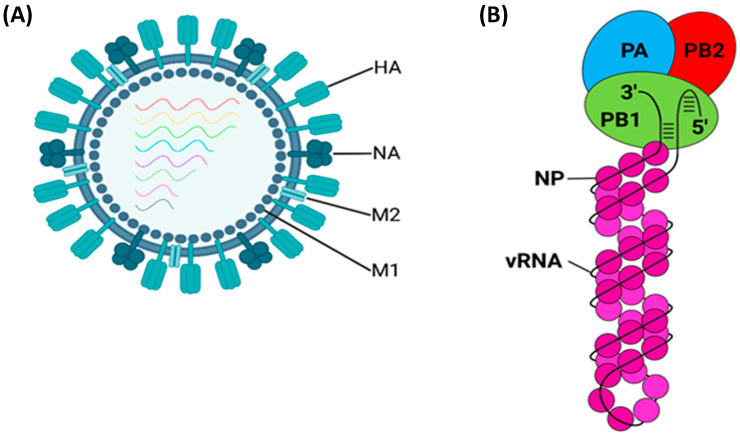
Simplified diagram of a (**A**) spherical IAV virion and (**B**) vRNP. (**A**) Spherical IAV virions are approximately 100–120 nm in diameter and comprise a viral envelope with the viral glycoproteins HA, NA, and M2 supported by an internal matrix layer of oligomerised M1 [16]. The segmented genome, here represented as coloured lines, is depicted more accurately in (**B**). (**B**) vRNPs contain the viral genome in a double helix of oligomerised NP capped by the heterotrimeric viral polymerase. Each vRNP is roughly 10 nm wide and 30–110 nm long [17]. This Figure depicts a right-handed double helix, but left-handed helices have also been reported [18]. Figure created with BioRender.com.

IAV virions adopt either spherical (approximately 100 nm diameter) or filamentous (approximately 100 nm wide and up to 20 µm long) morphologies that are observed in lab-adapted strains and clinically isolated samples, respectively [10,11]. Exactly why a spherical, as opposed to filamentous morphology, is adopted, and vice versa, remains unknown, but it likely results from delayed scission during the budding stage [12]. The viral envelope originates from the host plasma membrane and contains three transmembrane proteins: HA; NA; and matrix protein 2 (M2) [13,14,15]. Beneath the viral envelope is the matrix layer, a monolayer of oligomerised M1, which provides structural support [14]. Tethered to the matrix layer is the approximately 13,500 nucleotide (nt) viral genome, split into eight segments termed viral ribonucleoproteins (vRNPs) (Figure 1).

Each vRNP contains viral RNA (vRNA) wrapped around a double helical scaffold of oligomerised nucleoprotein (NP) with a loop at one end and a heterotrimeric viral polymerase at the other [19]; it seems likely but unconfirmed that the double helical scaffold is a mixture of right- and left-handed scaffolds [18]. The viral polymerase comprises polymerase basic 2 (PB2), PB1, and polymerase acidic (PA) and binds the vRNA 3′ and 5′ termini [20]. The vRNA termini are highly conserved and partially complementary, forming a base-paired duplex when bound to the polymerase [20].

**Figure 2:**
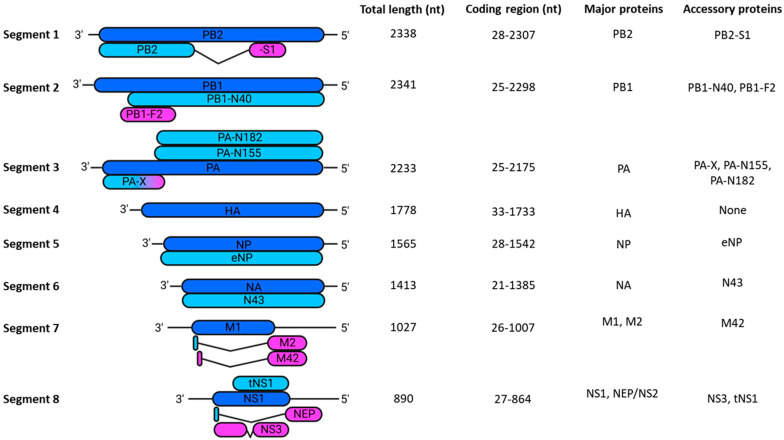
Schematic of the IAV genome. Each vRNA segment is displayed in anti-sense orientation with boxes representing coding regions in the corresponding viral mRNA. Dark blue represents primary products in frame 1; light blue represents secondary products in frame 1; magenta represents secondary products in frame 2, and diagonal lines represent mRNA splicing [21]. Nucleotide ranges shown are those for A/Puerto Rico/8/1934 (H1N1); the exact range varies by strain. Figure created with BioRender.com.

In total, there are eight vRNPs code for 10 major proteins essential for viral replication: PB2; PB1; PA; HA; NP; NA; M1; M2; non-structural protein 1 (NS1); and nuclear export protein (NEP, formerly NS2). The genome also encodes up to 11 non-essential accessory proteins: PB2-S1; PB1-N40; PB1-F2; PA-X; PA-N155; PA-N182; eNP; NA43; M42; NS3; and tNS1 (Figure 2) [21]. While the function of some accessory proteins remains unknown, most suppress the host’s innate immune response through a diverse array of mechanisms [21]. This important but inessential nature, coupled with their non-universal expression, justifies their exclusion from this review.

**Figure 3:**
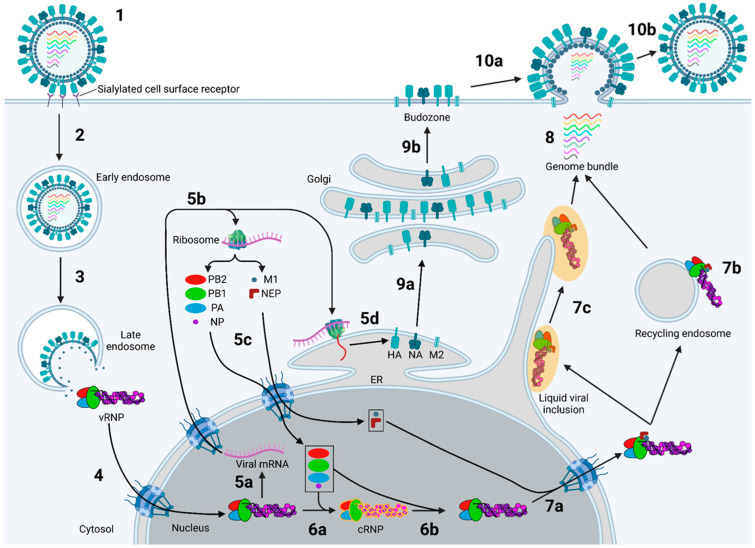
Overview of IAV replication. (**1**) IAV virions comprise a viral membrane containing HA, NA, and M2 supported by an inner matrix layer of oligomerised M1 [16]. The viral genome is split into eight vRNPs, represented as coloured lines for simplicity [19]. IAV virions can also be filamentous [10], but for clarity, this Figure depicts a spherical virion. (**2**) The replication cycle starts with the binding of HA to sialylated cell surface receptors [24], causing the virion to enter the cell contained in an early endosome; clathrin-mediated endocytosis is the primary mode of entry, but other mechanisms exist [23]. (**3**) As the early endosome matures to a late endosome, the corresponding drop in lumenal pH releases the HA fusion peptide, which buries itself in the endosomal membrane [22,37]; subsequent refolding of the extended HA intermediate fuses the viral and endosomal membranes, releasing the genome into the cytosol [22]. While only one vRNP is shown for simplicity, in reality, all eight are released as one bundle [25]. (**4**) The vRNP bundle is imported into the nucleus via the classical importin pathway [25]. (**5a**) The heterotrimeric viral polymerase on the vRNP snatches the first 10–13 nt from capped host mRNA and uses it and a vRNA template to transcribe viral mRNA [26,38,39] (**5b**), which is then exported and translated [40]. (**5c**) Cytosolic viral proteins (PB2, PB1, PA, NP, M1, and NEP) are imported into the nucleus [16]. (**5d**) Transmembrane viral proteins (HA, NA, M2) are translated into the ER [41]. The accessory proteins are omitted from this Figure for clarity. (**6a**) The vRNP-resident polymerase replicates a vRNA template into cRNA, which binds the nascently imported PB2, PB1, PA, and NP to form a cRNP [42]. (**6b**) The cRNP is similarly but not identically replicated into progeny vRNPs [27]. (**7a**) The progeny vRNPs are exported from the nucleus with the aid of M1 and NEP [30] before being trafficked to the plasma membrane either in (**7b**) recycling endosomes returning to the cell surface [29] or (**7c**) liquid viral inclusions that form and travel along a modified ER [28]. (**8**) The vRNPs form a fully assembled genome bundle en route to the plasma membrane [31]. (**9a**) The viral transmembrane proteins are translated into the ER membrane and trafficked to the Golgi, where HA is activated by proteolytic cleavage [16], (**9b**) and then to lipid rafts in the plasma membrane, which coalesce into a viral budozone, from which progeny virions bud [34,43]. (**10a**) The viral transmembrane proteins induce budding, with the budozone membrane being incorporated into the growing virion [44]. Cytoplasmic M1 oligomerises into a matrix layer beneath the membrane, and the fully assembled genome bundle is recruited into the tip of the budding virion [33,45]. (**10b**) Once a sufficient amount of budozone has been incorporated, M2-mediated scission separates the viral and plasma membrane, and the sialidase activity of NA releases the nascent virion from sialylated cell surface receptors [12,36]. Figure created with BioRender.com.

Replication of the genome involves several steps summarised in Figure 3. After binding to sialylated surface receptors, the virion is endocytosed and fuses with late endosomes to release the vRNPs into the cytosol [22,23,24]. Unusually for RNA viruses, IAV replicates in the nucleus and is imported via the classical importin pathway [25]. The vRNP-resident polymerase cap snatches nascent host mRNA to transcribe positive-sense viral mRNA, which is exported and translated [26]. The same vRNP then undergoes primary genomic replication, synthesising positive-sense complementary RNA (cRNA), which is swiftly captured by the nascently translated proteins to form a complementary RNP (cRNP); the cRNPs then undergo secondary replication to form progeny vRNPs [27]. While both viral mRNA and cRNA are positive-sense copies of the vRNA genome, there are several notable differences, including the lack of a 5′ 7-methylguanylated (m7G) cap and poly(A) tail in cRNA [20]. The progeny vRNPs are exported from the nucleus and trafficked to the plasma membrane via a combination of recycling endosomes and a modified endoplasmic reticulum (ER), forming a bundle with all eight segments while en route [28,29,30,31,32]. This fully assembled genome is loaded into the budding virion with the transmembrane viral proteins having previously been trafficked to the plasma membrane [33,34]. M2 induces scission of the viral and plasma membranes, after which the sialidase activity of NA releases the progeny virion from the cell surface [35,36].

### 1.1. Overview of Essential IAV Proteins

#### 1.1.1. Polymerase

**Figure 4:**
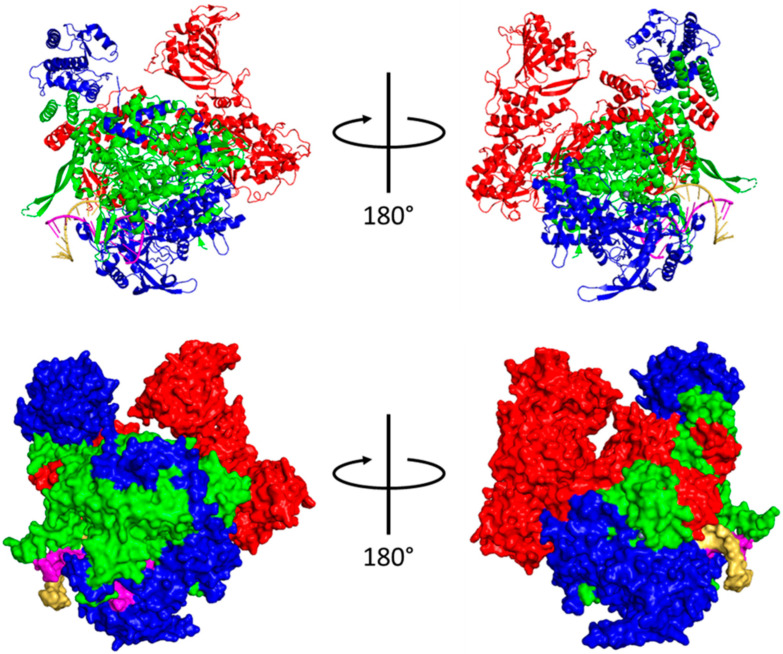
Structure of the A/little yellow-shouldered bat/Guatemala/060/2010 (H17N10) polymerase. Ribbon (**top**) and surface (**bottom**) structure. PB2 in red; PB1 in green; PA in blue; vRNA 5′ nt 1–16 in pink; vRNA 3′ nt 1–12 in yellow. PB2 residues 742–760 and PB1 residues 755–756 are absent. Structure rendered in PyMol from PBD 4WSB [47].

The IAV polymerase is an approximately 270 kDa heterotrimer containing PB2, PB1, and PA, which binds the v/cRNA 3′ and 5′ termini and transcribes and replicates the viral genome [46]. IAV polymerase adopts a U shape approximately 155 × 100 × 75 Å high, wide and deep, respectively (Figure 4) [46]. The three subunits are held together by extensive and reversible hydrophobic and polar contacts, which give the polymerase an extreme degree of conformational flexibility [46].

**Figure 5:**
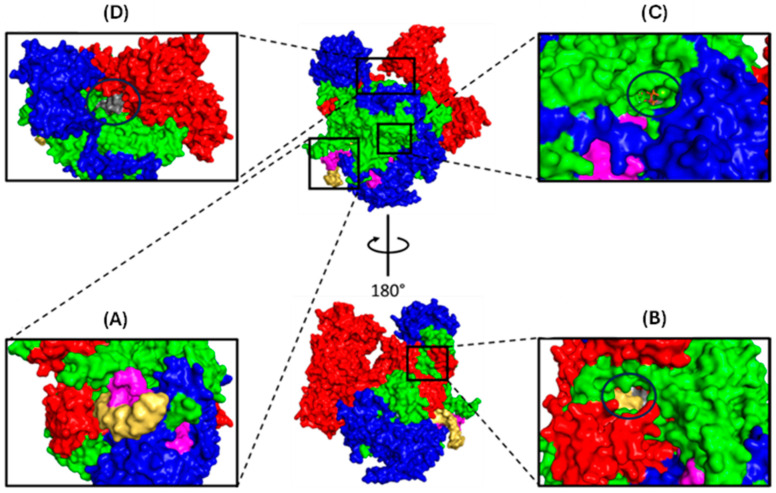
Location and structure of the template (**A**) entrance and (**B**) exit tunnels, the (**C**) NTP entrance tunnel, and (**D**) product exit tunnel. PB2 in red; PB1 in green; PA in blue; vRNA 5′ terminus in pink; vRNA 3′ terminus in yellow. (**A**) The template entrance tunnel, here occupied by vRNA, contains conserved residues from PB2, PB1, and PA [47]. Structure rendered in PyMol from PDB 4WSB [47]. (**B**) The template exit tunnel, comprising PB2 and PB1, is only formed after transcription initiation [48]. Structure rendered in PyMol from PDB 6QCT [48]. (**C**) Incoming NTPs enter the polymerase active site cavity through a tunnel formed by the PB1 fingertips and palm domain. Structure rendered in PyMol from PDB 6QCV [48]. (**D**) The product strand (grey) leaves the polymerase core between the PA-endonuclease and PB2 cap-binding domains. Structure rendered in PyMol from PDB 6QCV [48]. The structure in panel A is from A/little yellow-shouldered bat/Guatemala/060/2010 (H17N10). The structures in panels B-D are from B/Memphis/13/03.

The central portion of the polymerase comprises PB2-N, PB1, and PA-C. The two ‘arms’ are tipped by the PB2 cap-binding and PA-endonuclease domains, which are flexibly linked to the polymerase core and can adopt a wide array of conformations [47]. Most of the polymerase body comprises PB1, which forms a right-handed RNA-dependent RNA polymerase (RdRp) with fingers, palm, and thumb subdomains [46]. The catalytic core of the RdRp is accessible through template and NTP entrance tunnels and template and product exit tunnels (Figure 5).

#### 1.1.2. Polymerase Basic 2 (PB2)

**Figure 6:**
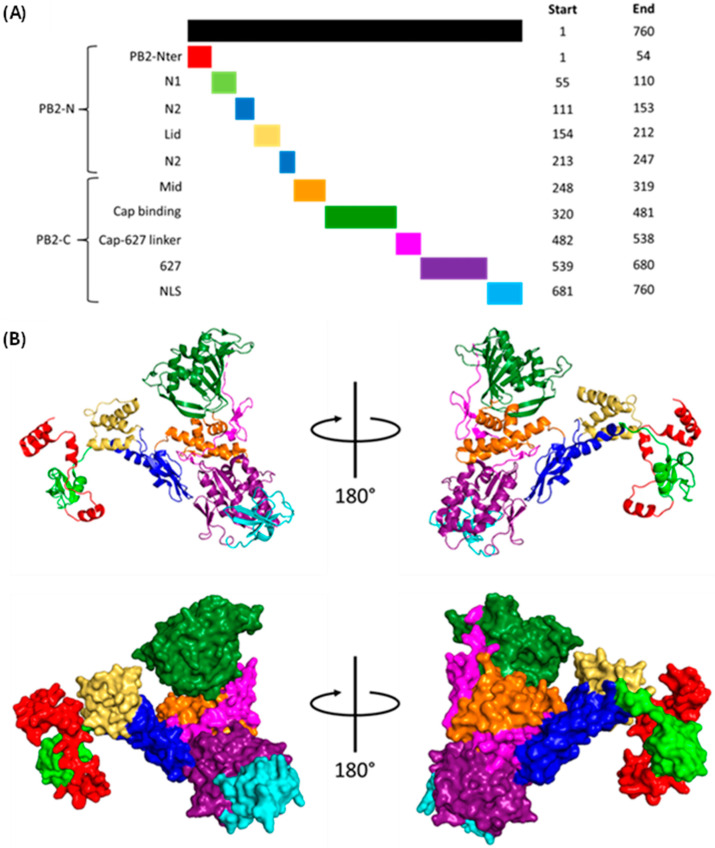
Structure of A/little yellow-shouldered bat/Guatemala/060/2010 (H17N10) PB2. (**A**) Primary structure of PB2 coloured by domain. PB2-Nter (residues 1–54) in red; N1 (55–110) in light green; N2 (111–153 and 213–247) in dark blue; lid domain (154–212) in yellow; mid-domain (248–319) in orange; cap-binding domain (320–481) in dark green; cap-627 linker domain (482–538) in pink; 627 domain (539–680) in purple; NLS domain (681–760) in light blue [47]. (**B**) Ribbon (top) and surface (bottom) structure of PB2. Domains coloured according to (**A**). Residues 742–760 are absent. Structure rendered in PyMol from PBD 4WSB [47].

PB2 weighs over 86 kDa and is split into an N-terminal third (PB2-N) and C-terminal two-thirds (PB2-C), both of which contain several domains [47] (Figure 6). PB2-N comprises the PB2-Nter, -N1, -N2 and helical lid domains. PB2-N1 and -N2 form hydrophobic contacts with PB1 C-ext and thumb domains and guide the vRNA 3′ terminus into the active site [47]. The PB2 helical lid contacts the PA endonuclease and PB2 cap-binding domains. PB2-C forms an arc containing the mid, cap-binding, cap-627 linker, 627, and NLS domains [47]. The mid domain forms stabilising interactions with the 627-linker; the cap-binding domain captures m7G caps; the highly flexible cap-627 linker joins the cap-binding domain to the polymerase body and permits its rotation by up to 70°; the 627 domain is involved in replication and species specificity; the NLS domain contains a bipartite nuclear localisation signal (NLS), which permits PB2 nuclear import via the classical importin pathway [46,47].

#### 1.1.3. Polymerase Basic 1 (PB1)

**Figure 7:**
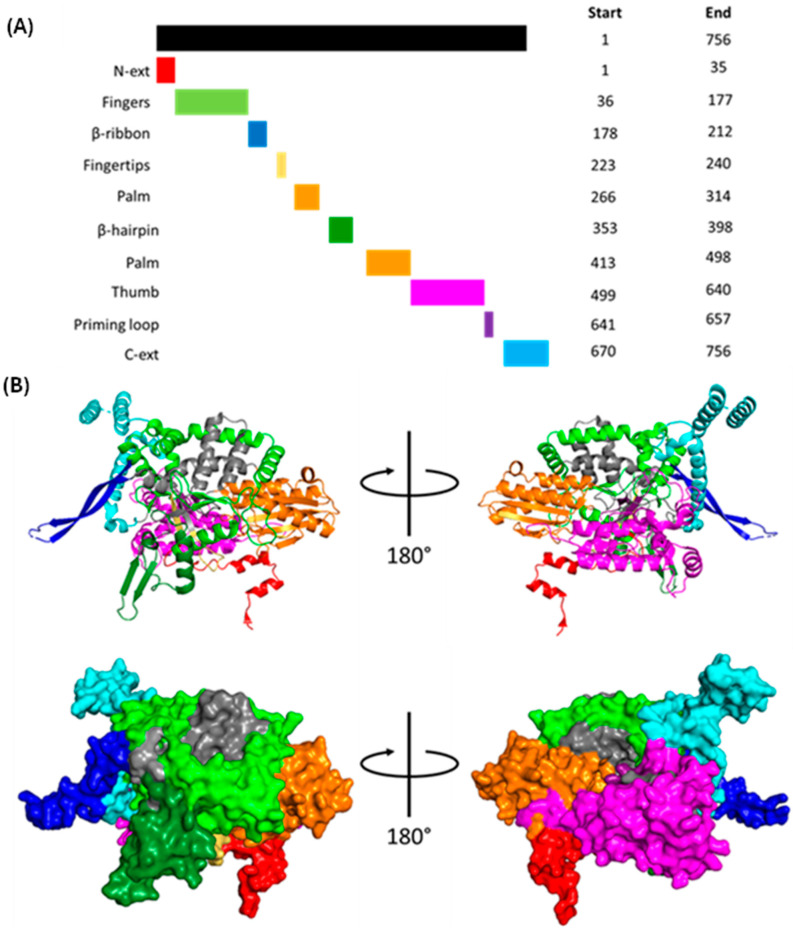
Structure of A/little yellow-shouldered bat/Guatemala/060/2010 (H17N10) PB1. (**A**) Primary structure of PB1 coloured by domain. N-ext (residues 1–35) in red; fingers (36–177) in light green; β-ribbon (178–212) in dark blue; fingertips (223–240) in yellow; palm domain (266–314 and 413–498) in orange; β-hairpin (353–398) in dark green; thumb domain (499–640) in pink; priming loop (641–657) in purple; C-ext (670–756) in light blue. Linker regions displayed in grey [47]. (**B**) Ribbon (top) and surface (bottom) structure of PB1 with domains coloured according to (**A**). Residues 755–756 are absent. Structure rendered in PyMol from PBD 4WSB [47].

The approximately 87 kDa PB1 forms the bulk of the polymerase body and contains nine domains: N-ext; fingers; β-ribbon; fingertips; palm; β-hairpin; thumb; priming loop; and C-ext (Figure 7). The N-ext domain interfaces with PA-C; the priming loop has important roles in transcription and replication; the β-ribbon contains the NLSs; the β-hairpin (along with the PA-C and PA-arch domains) forms the binding cleft for the vRNA 5′ terminus; the C-ext binds both the vRNA 3′ terminus and the PB2-N domain [47]. The fingers, β-ribbon, fingertips, palm, β-hairpin, thumb, and priming loop form the PB1 central region, the catalytic centre of the polymerase [47]. The PB1 catalytic core adopts a right-handed fold characteristic of RdRps with six highly conserved motifs: pre-A (also called F); A; B; C; D; and E. Motif A D305 and motif C D445 and D446 coordinate two catalytic divalent metal cations in the active site, and motifs C, D and E stabilise the incoming NTP [47].

#### 1.1.4. Polymerase Acidic (PA)

**Figure 8:**
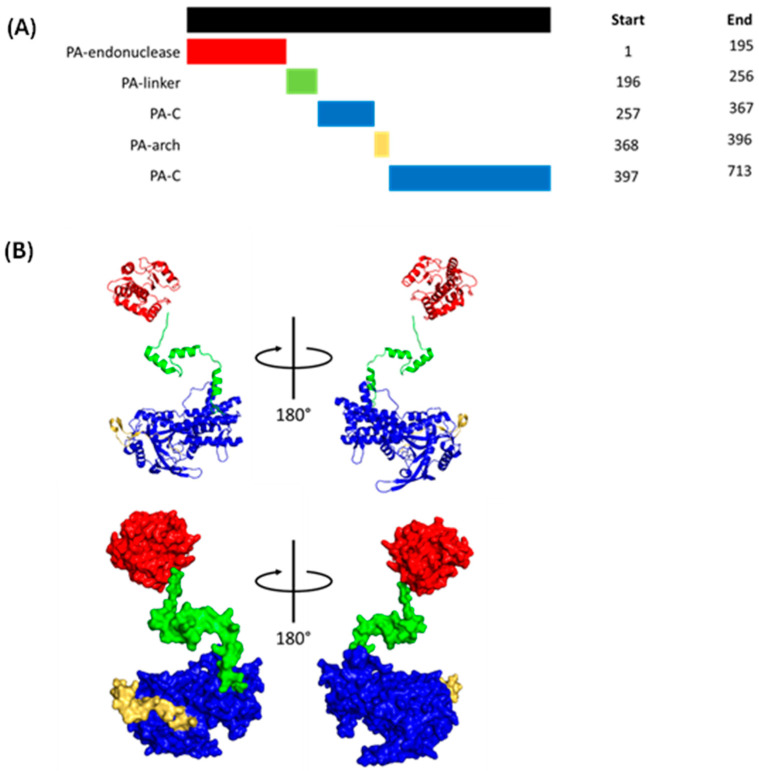
Structure of A/little yellow-shouldered bat/Guatemala/060/2010 (H17N10) PA. (**A**) Primary structure of PA coloured by domain. PA-endonuclease (residues 1–195) in red; PA-linker (196–256) in green; PA-C (257–367 and 397–713) in blue; PA-arch (368–396) in yellow [47]. (**B**) Ribbon (top) and surface (bottom) structure of PA. Domains coloured according to (**A**). Residues 186–200 and 715–724 are absent. Structure rendered in PyMol from PBD 4WSB [47].

PA weighs under 83 kDa and contains endonuclease, linker, C, and arch domains (Figure 8). The endonuclease domain faces the PB2 cap-binding domain, allowing for the endonuclease domain to cleave capped host mRNA captured by the PB2 cap-binding domain [47]. The linker domain bridges the endonuclease and C domains and forms polar and hydrophobic contacts with the PB1 fingers and palm domain [47]. The C domain binds the PB1 N-terminus to buttress the polymerase core, binds the host RNA polymerase II C-terminal domain (CTD) to initiate cap snatching, and forms one side of the v/cRNA 5′ terminus binding pocket [46]; the PB1 β-hairpin is partially inserted through the PA-arch domain to form the other side of the binding pocket [46,47].

#### 1.1.5. vRNA Promoter

**Figure 9:**
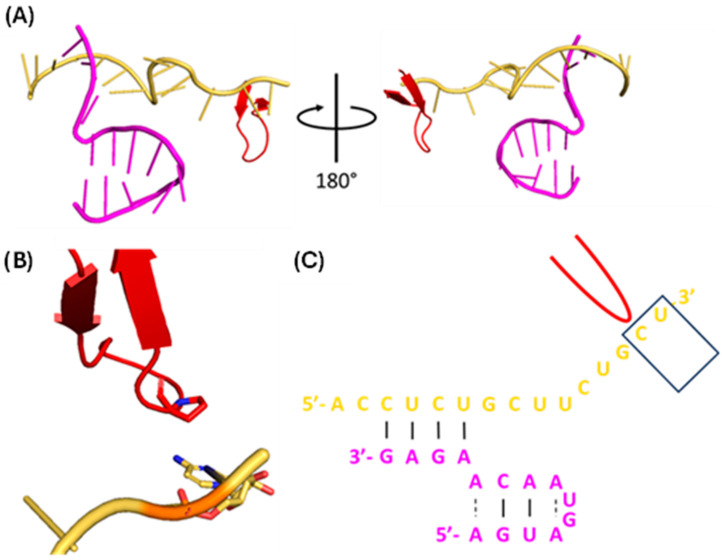
Ribbon structure (A + B) and simplified schematic (C) of the vRNA promoter stabilised in the active site. (**A**) vRNA 5′ terminus in pink, 3′ terminus in yellow, and PB1 priming loop in red. (**B**) Close-up of the vRNA-priming loop interaction. P651 of the priming loop (PB1 residues 641–657) interacts with vRNA 3′ U2, stabilising it in the polymerase active site [48]. (**C**) Colours same as in (**A**). Black lines represent complementary base pairs. Dashed lines represent non-complementary base pairs. The black box represents the location of the polymerase active site. Structure rendered in PyMol from PDB 6T0N [39].

The vRNA 5′ terminus (5′-AGUAGUAACAAGAGG-3′) and 3′ terminus (5′-UAUACCUCUGCUUCUGCU-3′) are highly conserved and quasi-complementary, forming a duplex promoter with multiple inter- and intra-strand base pairs (Figure 9) [46]. When bound to the polymerase, vRNA 5′ nt 1–10 forms the 5′ hook, a four-base pair (bp) stem-loop that forms extensive polar interactions with a pocket formed at the PB1-PA interface [46]. vRNA 3′ nt 1–9 and cRNA 3′ nt 1–11 are single-stranded, highly flexible and bind either the polymerase active site or a secondary binding site on the polymerase surface [48]. vRNA 3′ nt 10–13 and cRNA 3′ nt 12–15 form four inter-strand base pairs with v/cRNA 5′ nt 11–14 to create a duplex that maintains the pseudocircularity of the viral genome [46]. While the structure of the complete cRNA promoter remains unknown, the first 12 nt of the cRNA 5′ terminus are virtually identical to that of vRNA [49]; because of this, it is presumed that the cRNA and vRNA promoters have virtually identical secondary structures.

#### 1.1.6. Haemagglutinin (HA)

**Figure 10:**
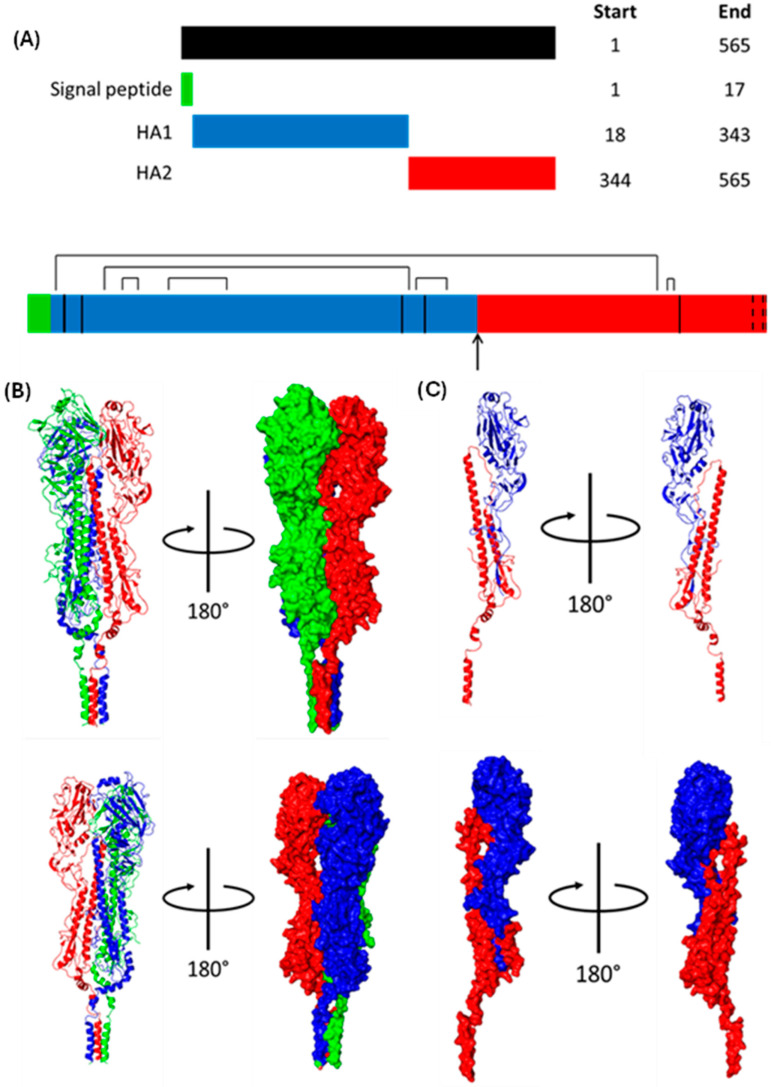
Structure of HA. (**A**) HA0 primary structure highlighting key regions (top) and post-translational modifications (PTMs, bottom). Signal peptide (residues 1–17) in green; HA1 (18–343) in red; HA2 (344–565) in blue. Black bars represent disulphide bonds (intra-HA1 disulphide bonds C59-291, C72-84, C107-152, C295-319; intra-HA2 disulphide bonds C487-491; inter-HA1-HA2 disulphide bonds C21-480). Black lines represent N-linked glycosylation sites (N27, N28, N40, N285, N303, and N497). Dashed lines indicate lipidation sites (C554, C561, C565). Arrow indicates location of cleavage site. PTM residues from labelling of UniProt entry P03452 (A/Puerto Rico/8/1934 (H1N1)) by UniRule HAMAP-Rule: MF_04072 following immature H1 numbering [54,55]. Ribbon (top) and surface (bottom) structure of the A/duck/Alberta/35/76 (H1N1) HA (**B**) trimer and (**C**) monomer. (**C**) Coloured as per (**A**). Residues 531–566 are absent. Structure rendered in PyMol from PDB 6HJQ [51].

HA weighs approximately 63 kDa and is initially translated as HA0, one continuous polypeptide with an ER signal sequence at the N-terminus that is cleaved shortly after translation [50] (Figure 10). HA0 homotrimerises post-translation before being trafficked to the Golgi, where each monomer is proteolytically cleaved into an HA1-HA2 dimer linked by a disulphide bond [51,52,53]. HA1 mediates cell entry by binding 5-acetylneuraminic acid (Neu5Ac), whereas HA2 is responsible for viral–endosomal membrane fusion [24,37]. The mature HA trimers are trafficked to the cell surface before being incorporated into the viral membrane [44]. Unless otherwise stated, the numbering of HA residues henceforth follows the mature H3 numbering system [54].

#### 1.1.7. Nucleoprotein (NP)

NP weighs approximately 56 kDa and adopts a crescent shape with a head domain, body domain, and tail loop [56]. It encapsidates v/cRNA and mediates the nuclear import of parent vRNPs through three NLSs [57,58]. Insertion of the tail loop into an adjacent NP monomer permits homo-oligomerisation, thus forming a scaffold to which most of the viral genome binds [19,59]. The binding of v/cRNA to NP protects it from degradation by host cell nucleases [60].

#### 1.1.8. Neuraminidase (NA)

NA weighs almost 50 kDa and contains a cytoplasmic tail, transmembrane domain, stalk domain, and catalytic head domain [61]. The cytoplasmic tail aids M1 and vRNP recruitment to budding virions; the transmembrane domain is predicted to be an α-helix, which ensures NA membrane association; the catalytic head domain forms a six-bladed propellor, with each propellor containing four anti-parallel β-sheets stabilised by disulphide bonds [61]. NA cleaves sialic acid from the underlying glycan that connects HA from nascent progeny IAV virions to the cell surface, freeing the virion [62].

#### 1.1.9. Matrix Protein 1 (M1)

M1 is approximately 28 kDa and comprises an N-terminal domain (NTD) and CTD joined by a short linker [63,64]. After recruitment to the plasma membrane by the HA and NA cytoplasmic tails, it oligomerises into helical filaments, which provide structural support and recruit progeny vRNPs [63,65].

#### 1.1.10. Matrix Protein 2 (M2)

M2, weighing over 11 kDa, is produced by alternative splicing of M1 mRNA and forms a homotetramer of two disulphide-linked dimers [66,67]. Each monomer contains an N-terminal ectodomain, transmembrane domain, amphipathic helix, and cytoplasmic tail [68]. M2 has important roles in viral–endosomal membrane fusion [68] and scission [69].

#### 1.1.11. Nuclear Export Protein (NEP)

NEP (formerly NS2) is an approximately 14 kDa protein produced by alternative splicing of NS1 mRNA and comprises an NTD and CTD [70,71,72,73]. Two N-terminal nuclear export signals (NESs) mediate the nuclear export of progeny vRNPs [30,74].

## 2. Binding and Endocytosis

### 2.1. Sialic Acid

**Figure 11:**
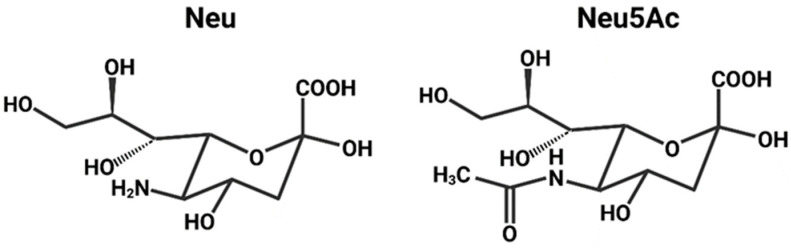
Structure of neuraminic acid (Neu, left) and 5-acetylneuraminic acid (Neu5Ac, right). Neu5Ac is the most common neuraminic acid derivative [75] and is the receptor for IAV HA [81]. Figure created with BioRender.com.

**Figure 12:**
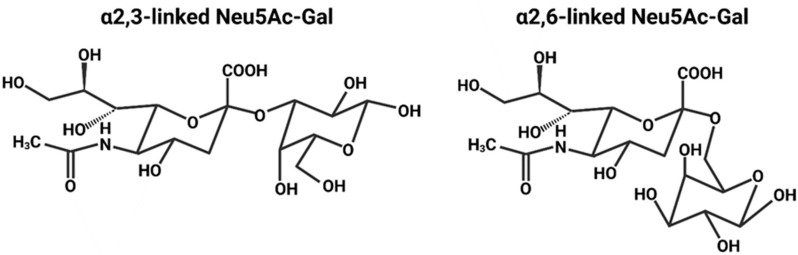
Structure of α2,3-linked (left) and α2,6-linked (right) Neu5Ac-Gal. In cells, this disaccharide is found at the end of a long oligosaccharide chain [77]. Figure created with BioRender.com.

HA mediates cell entry by binding Neu5Ac, a type of sialic acid [24]. The sialic acids are nine-carbon amino sugars with an amine group on the carbon-5 [75]. Neuraminic acid is one such example, and acetylation of the amine group yields Neu5Ac (Figure 11) [76]. Neu5Ac is the most common sialic acid in humans and is usually the terminal sugar residue on N-linked glycans, oligosaccharide chains linked to the N of an N-X-S/T sequence in a protein [77,78,79,80]. Neu5Ac is linked to the penultimate sugar residue of a glycan (usually galactose) via an α2,3 or α2,6 bond (Figure 12) [78,79].

The HA of any particular strain usually displays a preference for either α2,3- or α2,6-linked Neu5Ac. Avian-adapted strains typically favour α2,3-linked Neu5Ac due to its overwhelming presence in the intestines, the major site for avian IAV infection [76]. Human-adapted strains usually prefer α2,6-linked Neu5Ac, which is displayed by epithelial cells of the upper respiratory tract; the accessible location of these cells enables efficient human-to-human transmission via aerosol droplets [2]. In humans, α2,3-linked Neu5Ac is typically displayed by cells of the lower respiratory tract; consequently, when humans are infected with an avian-adapted strain, which prefers α2,3-linked Neu5Ac, the infection is usually localised to the lower respiratory tract and, thus, direct human-to-human transmission is far less efficient [2]. Pig tracheae contain both α2,3- and α2,6-linked Neu5Ac, meaning that both avian- and human-adapted strains can establish an infection and be efficiently transmitted [76,82]. This susceptibility enables pigs co-infected with avian- and human-adapted strains to serve as ‘mixing vessels’, which generate and transmit reassortment strains to humans [82,83,84].

### 2.2. HA1 Structure and Receptor Binding

**Figure 13:**
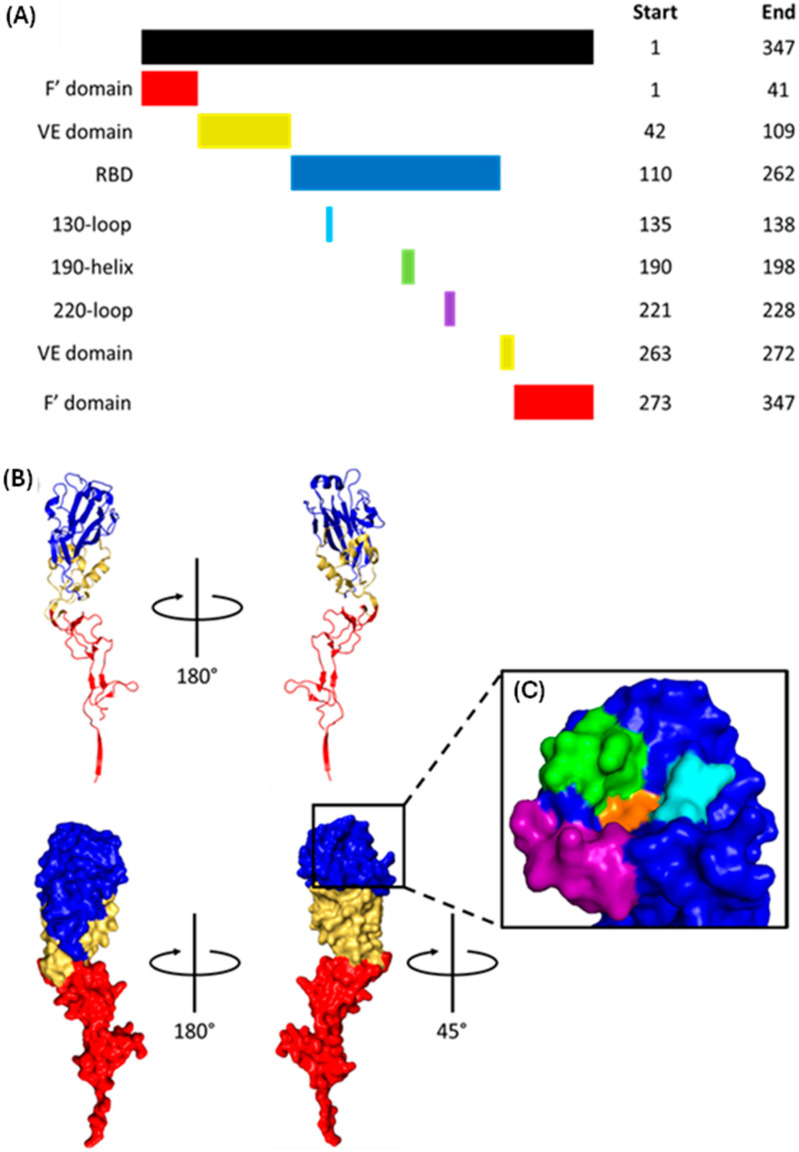
Structure of the A/Duck/Singapore/3/97 (H5N3) HA1 monomer. (**A**) Primary structure of HA1 coloured by domain. Fusion (F’) domain (residues 1–41 and 273–330) in red; vestigial esterase (VE) domain (42–109 and 263–272) in yellow; receptor binding domain (RBD, 110–262) in dark blue [86]. The 130-loop (135–138, light blue), 190-helix (190–198, green), and 220-loop (221–228, purple) make up the sides of the receptor binding site (RBS) [87]. Residue ranges follow mature H3 numbering [54]. (**B**) Ribbon (**top**) and surface (**bottom**) structure of the HA1 monomer coloured as per (**A**) with the 130-loop, 190-helix, and 220-loop coloured dark blue. Residues 322–347 are absent. (**C**) Close-up of the HA1 RBD highlighting the RBS. The 130-loop, 190-helix, and 220-loop are coloured as per (**A**) with Y98, W153, and H183 coloured orange [24,78,80]. Structure rendered in PyMol from PDB 1JSN [88].

HA1, the N-terminal component of mature HA, comprises a fusion domain, vestigial esterase domain, and receptor binding domain (RBD). The RBD contains the receptor binding site (RBS), a cleft with walls made from the 130-loop, 190-helix and 220-loop, and a base formed by Y98, W153, and H183 (Figure 13) [24,78,80,85].

**Figure 14:**
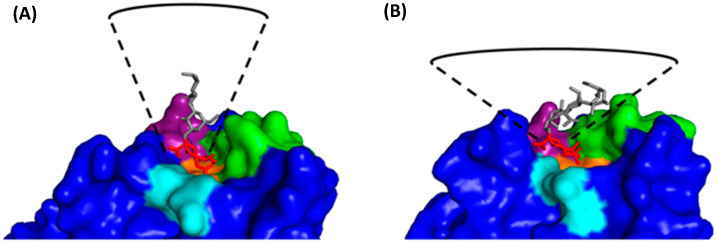
Comparison of the binding of (**A**) α2,3-linked and (**B**) α2,6-linked Neu5Ac to HA. (**A**) Binding of α2,3-linked Neu5Ac to avian-adapted A/Duck/Singapore/3/97 (H5N3) HA. (**B**) Binding of α2,6-linked Neu5Ac to human-adapted A/Singapore/1/57 (H2N2) HA. The black boxes represent the cone-like and umbrella-like volumes occupied by the α2,3-linked and α2,6-linked glycans, respectively. Both structures are coloured following Figure 13C with Neu5Ac in red and the remaining glycan in grey. Structures rendered in PyMol from PDB 1JSN [88] and PDB 2WR7 [93].

HA binds to α2,3- and α2,6-linked Neu5Ac via fundamentally different mechanisms (Figure 14) [89]. When binding to avian-adapted HA, α2,3-linked Neu5Ac adopts an extended conformation and occupies a cone-like volume around the RBS [85,90]. Several highly conserved RBS residues interact with the glycan base (Neu5Ac-Gal), with the restrictive cone-like conformation preventing binding to the rest of the glycan [85]. When α2,6-linked Neu5Ac binds to human-adapted HA, the glycan folds back on itself and adopts a much wider umbrella-like volume [85]. The kink introduced by the α2,6 bond means HA adapted towards α2,6-linked Neu5Ac must have a slightly larger RBS [91]. The glycan base is recognised by the same residues as for α2,3-linked Neu5Ac, but the much wider umbrella-like conformation allows the remaining sugar residues to make several charged and polar interactions outside the RBS [85,92].

Species specificity is provided by residues 226 and 228; avian-adapted HA has Q226 and G228, whereas human-adapted HA has L226 and S228 [78]. Simultaneous Q226L and G228S mutations widen the RBS, allowing for the larger base of the human α2,6-linked Neu5Ac to bind [91].

### 2.3. Clathrin-Mediated Endocytosis

**Figure 15:**
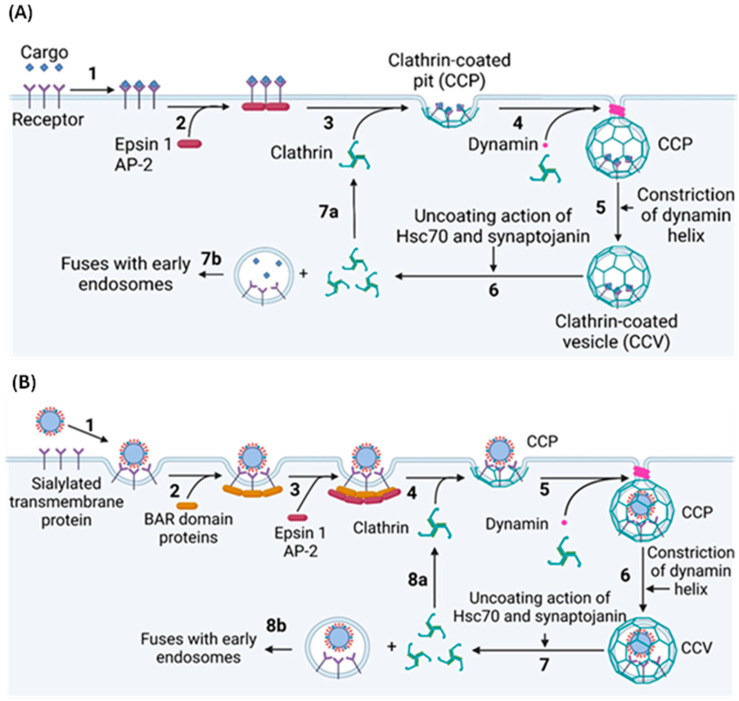
Clathrin-mediated endocytosis (CME) of (**A**) normal cargo and (**B**) spherical IAV virions. (**A**) **1:** CME starts with the binding of extracellular cargo to endocytic receptors [98]. **2:** This binding exposes a cytosolic endocytosis motif, permitting the binding of adaptor proteins such as Epsin 1 and AP-2 [97]. **3:** These adaptor proteins induce clathrin polymerisation at the site of cargo binding, thus forming a spherical CCP [104]. The adaptor proteins are omitted for clarity. **4:** Once CCP formation is complete, dynamin forms an oligomeric helix around the CCP neck [106]. **5:** Energy from GTP hydrolysis constricts the helix, severing the neck to release a CCV into the cytosol [105]. **6:** Steric forces introduced by Hsc70 and disruption of membrane–protein interactions by synaptojanin remove the clathrin lattice from the CCV [103,109]. **7a:** The now monomeric clathrin polymerises at a new CCP. **7b:** The endocytosed vesicle fuses with early endosomes [103]. (**B**) **1:** The binding of multiple HA trimers to host transmembrane proteins may cause them to cluster and form a region of localised membrane curvature [23]. **2:** This localised membrane curvature could be sensed by various BAR domain proteins, **3:** which might recruit the adaptor proteins Epsin 1 and AP-2 and from here, the process is like CME of ordinary cargo [23]. **4:** Clathrin is recruited by the adaptor proteins to form CCPs [63,97,104]. The adaptor proteins are omitted for clarity. **5:** Dynamin forms an oligomeric helix around the CCV neck [106]. **6:** This helix constricts to release the CCV into the cytosol [105], where **7:** Hsc70 and synaptojanin disassemble the clathrin lattice [103]. **8a:** The monomeric clathrin is recycled, and **8b:** the uncoated vesicle containing a spherical IAV virion fuses with early endosomes, beginning the next step of IAV replication [16]. Figure created with BioRender.com.

Approximately 70% of IAV virions entering the cell do by clathrin-mediated endocytosis (CME), the most common form of endocytosis, which involves the formation, scission, and uncoating of clathrin-coated pits (CCPs) (Figure 15) [23,94]. CCPs form randomly across the plasma membrane, but most collapse soon after formation unless stabilised by the binding of extracellular cargo to transmembrane endocytosis receptors [95]. This binding exposes cytosolic motifs, which are recognised by adaptor proteins, which, in turn, recruit clathrin to the site of cargo binding, initiating the CCP formation [96,97,98]. Adaptor Protein 2 (AP2) is the most common clathrin adaptor protein, but Epsin 1 is most relevant to the CME of IAV virions [99,100]. AP2 and Epsin 1 are initially recruited to the plasma membrane by binding phosphatidylinositol-4,5-bisphosphate (PI(4,5)P_2_), a phospholipid enriched in the plasma membrane with important roles in several signalling pathways, including CME [97,101,102]. The binding of AP2 and Epsin 1 to PI(4,5)P_2_ exposes the endocytosis motif binding site, localising the adaptor proteins to the activated endocytosis receptor. Clathrin polymerises into a 100 nm wide spherical lattice, which induces membrane curvature by imposing its shape onto the underlying membrane [103,104]. This, along with various adaptor proteins, provides the energy required to deform the plasma membrane [103]. Once the clathrin lattice is fully assembled, it forms a CCP which remains connected to the plasma membrane via a small neck, the scission of which is required to release the CCP into the cytosol as a clathrin-coated vesicle (CCV) [103]. Scission is performed by dynamin, a GTPase that forms helical oligomers around the CCP neck. GTP hydrolysis induces a conformational change that constricts the helix, pinching and ultimately severing the neck to separate the CCV from the plasma membrane [105,106].

Before the CCV enters the intracellular trafficking system, the clathrin coat is removed by the ATPase Hsc70 and the phosphatase synaptojanin [103]. Hsc70 is recruited to the CCV immediately post-scission and uses energy from ATP hydrolysis to drive clathrin depolymerisation, likely by introducing disruptive forces into the vertices of the lattice [103,107,108,109,110]. Synaptojanin dephosphorylates PI(4,5)P_2_, which likely causes CCV uncoating by destabilising numerous membrane–coat interactions, although the exact mechanism remains elusive [103].

While CME in uninfected cells requires dedicated protein receptors, HA binds to terminal Neu5Ac and not a specific endocytosis receptor, raising the question of how binding induces CME [81,103]. It is possible that the binding of multiple HA trimers in a concentrated region on the plasma membrane clusters host transmembrane proteins to induce localised membrane curvature. This curvature can be detected by BAR domain proteins, some of which recruit clathrin either directly or through adaptor proteins [23]. This multivalent binding may also cause the localised clustering of receptor tyrosine kinases (RTKs), thereby activating the intracellular signals necessary for IAV cell entry [111].

### 2.4. Clathrin-Independent Endocytosis

Filamentous IAV virions are too large for uptake by CME, instead entering cells through the macropinocytosis [112]. Macropinocytosis is the primary mechanism used to non-selectively internalise extracellular fluids via the actin-dependent formation of plasma membrane ruffles that protrude from the cell surface [113]. Most ruffles retreat back into the cell, but some fold back and fuse with the plasma membrane to form a macropinosome up to 5 µm wide [114]. Once the macropinosome enters the cytosol, it matures similarly to endosomes, gradually shrinking and acidifying before fusing with lysosomes [113,114]. The decreasing pH of the maturing macropinosome induces drastic membrane curvature, which fragments the filamentous virions [112]. As mentioned above, the multivalent binding of HA can cause localised clustering and activation of RTKs [111]. This is particularly relevant to macropinocytosis as RTKs activate Rab5, a protein involved in actin remodelling [115]; given that actin remodelling is an essential feature of macropinocytosis [112], it seems plausible to propose that RTK clustering is a possible method of HA-induced macropinocytosis.

## 3. Fusion of the Viral and Endosomal Membranes

Immediately post-endocytosis, the IAV virion is enclosed in a primary endocytic vesicle; a drop in pH is required to release the viral genome into the cytosol [16]. This process starts with the fusion of the primary endocytic vesicle with early endosomes, mildly acidic vesicles, which serve as the main sorting station of the endocytic pathway [116]. As early endosomes accumulate more cargo, the membrane buds inwards and is pinched off. This and the continual lumenal acidification by V-ATPase form late endosomes with a pH of 6.0–4.8 [116,117,118]. While moving along microtubule tracks towards the perinuclear region, late endosomes fuse with lysosomes to form endolysosomes with a pH of roughly 4.5, which destroys any remaining lumenal material [116].

While endosome acidification is necessary for the proper functioning of the endocytic system, it is exploited by IAV to release the viral genome into the perinuclear region [16,117]. Genome release is reliant on several pH-dependent processes: virion interior acidification; viral–endosomal membrane fusion; matrix layer dissociation; and vRNP release [16,22].

### 3.1. Virion Acidification

Acidification of the virion interior is mediated by M2, a transmembrane homotetramer that transports up to 100 protons per second across the viral membrane [67,119,120]. The minimal proton conductance domain comprises a transmembrane helix (residues 26–46), which forms a narrow channel, and an amphipathic helix (residues 48–58), which forms a stabilising base [73,121]. H37 and W41 both form tetrads, which constrict the channel to a narrow neck, providing a proton selectivity filter and channel gate, respectively [122,123].

At neutral pH, the H37 and W41 tetrads are tightly packed. The low overall charge of the H37 tetrad reduces electrostatic repulsion and permits the formation of water wires from the channel entrance to the tetrad. Water wires are a mode of proton transport whereby a proton is transported along adjacent water molecules in a chain [124,125]. The hydrophobicity of W41 dehydrates the internal face of the channel gate, preventing the formation of internal water wires; this lack of connected water wires on both sides of the channel gate prevents proton conductance across the viral membrane at neutral pH [124,125].

When the pH drops to between 7.0 and 5.0 [126], H37 can become biprotonated [127]; biprotonation of two or more H37 residues forces the H37 tetrad apart through electrostatic repulsion, allowing for the formation of cation-π interactions between H37 and W41 [73,123,128]. This dilates the channel enough to hydrate the W41 tetrad and, thus, forms water wires on both sides of the channel gate [73,124,129]. The base formed by the amphipathic helices likely prevents the electrostatic repulsive forces from dissociating the homotetramer [121]. A proton is then transferred from a biprotonated H37 to the internal water wire. The loss of this proton reduces the charge of the H37 side chain and, thus, the overall electrostatic repulsion, returning the channel to its original conformation with a restricted and partially dehydrated neck that is incompatible with proton conductance [73].

### 3.2. Virial–Endosomal Membrane Fusion

**Figure 16:**
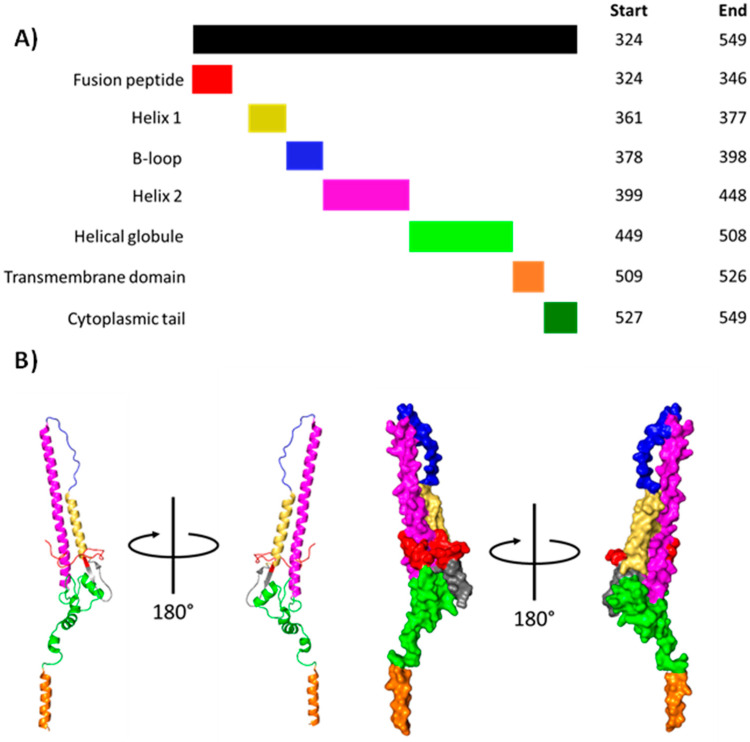
Structure of A/duck/Alberta/35/76 (H1N1) HA2 at neutral pH. (**A**) Primary structure of HA2 with key regions highlighted. Fusion peptide (residues 324–346) in red; helix 1 (361–377) in yellow; B-loop (378–398) in blue; helix 2 (399–448) in pink; helical globule (449–508) in light green; transmembrane domain (509–526) in orange; cytoplasmic tail (527–549) in dark green [51,130,135]. Residue ranges follow mature H3 numbering [54]. (**B**) Ribbon (left) and surface (right) structure of HA2 at neutral pH coloured as per (**A**). Residues 527–549 are absent. Structure rendered in PyMol from PDB 6HJQ [51].

Viral–endosomal membrane fusion is mediated by HA2, the C-terminal component of mature HA. HA2 comprises a fusion peptide, helix–loop–helix motif (itself containing helix 1, the B-loop and helix 2), helical bundle, transmembrane domain (TMD), and cytoplasmic tail (Figure 16) [51,130,131,132]. At neutral pH, the hydrophobic fusion peptide is buried in a cavity at the trimerisation interface [24,133,134].

**Figure 17:**
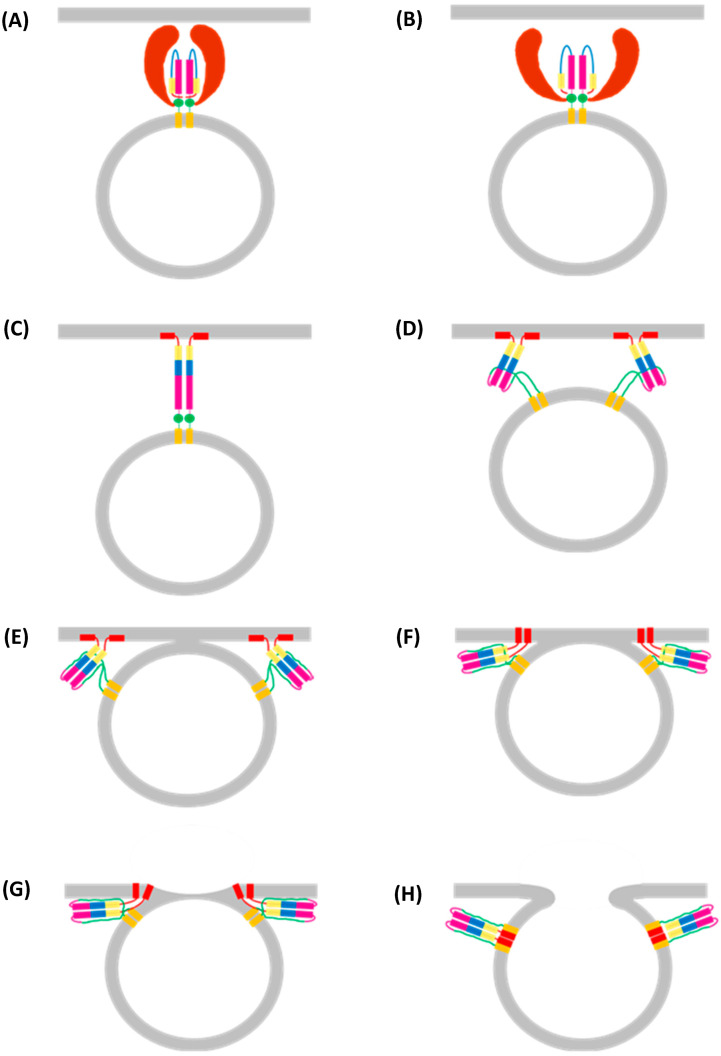
Mechanism of HA-mediated fusion of viral and endosomal membranes. (**A**) The HA1 subunit (brown) binds to sialic acid on cell surface receptors and induces the endocytosis of the virion into an endosome [136]. HA2 (fusion peptide in red; helix 1 in yellow; B-loop in blue; helix 2 in pink; helical globule in green; transmembrane domain in orange) remains enclosed by the HA1 head domain. For simplicity, only two HA1-HA2 dimers are shown; the presence of the other viral proteins and vRNPs are likewise omitted. (**B**) At sufficiently low pH, the protonation of multiple histidine residues causes electrostatic repulsion between the HA1 head domains, dilating the central pore [137,138]. Electrostatic repulsion between protonated residues also releases the fusion peptide from its binding cavity [133]. (**C**) Unrestricted by the HA1 head domain, the B-loop forms an α-helix continuous with helix 1 and helix 2, creating an extended coiled coil that places the fusion peptide approximately 100 Å away from the viral membrane [134,139,140]. The fusion peptide embeds itself into the endosomal membrane as a transmembrane helix or peripheral helical hairpin [131,141]. Panels (**C**–**E**) show the peripheral conformation; panels (**F**–**H**) show the transmembrane conformation. While the HA1 subunit remains attached to HA2 throughout the fusion process, it has been omitted from panels (**C**–**H**) for simplicity. (**D**) The helical globule and part of helix 2 melt and refold along the length of the extended coiled coil, bringing the endosomal and viral membranes closer together [142]. An additional HA trimer (also with only two HA2 subunits shown for clarity) is included to better convey how the conformational changes in HA2 drive viral–endosomal membrane fusion. (**E**) Once enough HA2 has been refolded, sufficient energy has been released to permit hemifusion of the outer viral and inner endosomal membranes, which forms a narrow hemifusion stalk [143]. (**F**) Displacement of lipid tails in the endosomal membrane by the fusion peptide is presumed to make them flow into the hemifusion stalk, expanding it into a hemifusion diaphragm [144]. (**G**) The HA2 fusion peptide and transmembrane domain presumably diffuse through the merged membrane; as they move towards each other, the transmembrane fusion peptide is assumed to recruit lipids from the outer endosomal membrane, causing an inwards bulge [144]. (**H**) The fusion peptides and transmembrane domains merge into a six-helix bundle, the energy of formation of which allows for the fusion of the inner viral and outer endosomal membranes [16]. With this, the fusion process is complete, and the viral genome enters the host cell cytoplasm after the dissociation of the M1 matrix layer [22,145]. The 3D structures of HA are available for the stages in panels (**A**–**C**,**H**) (PDB 6HJQ [51], 6Y5J [135], 6Y5K [146], and 1HTM [139], respectively); structures in other panels are inferred.

Viral–endosomal membrane fusion requires the dissociation of HA1, insertion of HA2 into the endosomal membrane, and refolding of the HA2 extended intermediate (Figure 17).

#### 3.2.1. HA1 Dissociation

Viral–endosomal membrane fusion requires the insertion of the fusion peptide into the endosomal membrane [147]. This, in turn, requires the outwards movement of the HA1 head domains relative to the central coiled coil [147,148,149,150]. HA1 dilation occurs at a pH under 5.8 and is achieved by the simultaneous protonation of key histidine residues located throughout the trimer, introducing electrostatic repulsion, which forces the HA1 head domains apart [135,137,138,147,149,150]. Dilation of the HA1 head domains widens the central cavity enough to allow water molecules to enter the central cavity, which presumably induces the necessary conformational changes in HA2 [138].

#### 3.2.2. Formation of the HA2 Extended Intermediate

Now unrestricted, the HA2 B-loop forms an α-helix continuous with helix 1 and helix 2. Such a change occurring in all three HA2 subunits forms an extended coiled-coil intermediate, which relocates the fusion peptide some 100 Å away from the viral membrane. Release of the fusion peptide from its binding cavity is presumed to also involve electrostatic repulsion resulting from the protonation of ionisable residues [134,151,152]. The highly hydrophobic fusion peptide buries itself in the endosomal membrane [139,145]. The exact structure it adopts is disputed, but the current consensus is either an extremely tight helical hairpin that lies parallel to the membrane plane [131] or a transmembrane helix [141].

#### 3.2.3. Refolding of the HA2 Extended Intermediate

Following the formation of the extended intermediate, the helical globule and an inner region of helix 2 are assumed to melt, losing all secondary structure. The putative melted HA2 C-terminus folds back along the remaining coiled coil [139]. This folding occurs via the ‘leash in a groove’ mechanism, with the melted ‘leash’ establishing several salt bridges, hydrophobic and cation-π interactions with the coiled coil ‘groove’, bringing the HA2 transmembrane domain and fusion peptide towards each other [142]. Because the transmembrane domain and fusion peptide remain embedded in the viral and endosomal membranes, respectively, this refolding brings both membranes closer [153].

The fusion of the viral and endosomal membranes is initially prevented by a high kinetic barrier [154]. Once enough of the melted ‘leash’ folds along the ‘groove’, the energy released overcomes this barrier, and the outer viral and inner endosomal membranes form a small hemifusion stalk [143,144]. Now that the two membranes have merged, the lipid tails displaced by the transmembrane domain and fusion peptide are forced into the hemifusion stalk, expanding it into a hemifusion diaphragm [144]. With the two membranes forming a wide interface, the transmembrane domains and fusion peptides diffuse through the membrane and merge into a six-helix bundle [16]. During this diffusion, the fusion peptide is assumed to adopt a transmembrane conformation; as it merges with the transmembrane domain, it pulls on the outer endosomal membrane, fusing it with the inner viral membrane to form a small fusion pore [144].

### 3.3. Matrix Layer Dissociation and vRNP Release

Concomitant to viral–endosomal membrane fusion is the dissociation of the matrix layer, which may render the viral membrane more flexible and is required to complete the fusion [155,156]. Without matrix layer dissociation, diffusion through and widening of the fusion pore is blocked by a 4 nm thick monolayer of helical filaments of oligomeric M1. vRNPs must also dissociate from M1 to be released into the cytosol [22]. Much like HA, the mechanism underlying matrix layer dissociation likely involves a conserved pH-sensing histidine cluster. When ionised, the histidine side chains introduce electrostatic repulsion, which destabilises the matrix layer and removes it from the viral membrane [157]. The matrix layer remains dissociated by forming a more stable multilayered structure [63]. While no direct evidence exists, it is plausible that the previously discussed histidine cluster and accompanying conformational changes at acidic pH are responsible for the release of vRNPs from M1 [63].

## 4. Nuclear Import of vRNPs

Because viral–endosomal membrane fusion occurs in late endosomes, which are trafficked to the perinuclear region, the vRNPs are released near the nucleus of the cytosol [116,158]. Owing to its highly sensitive nature, access to the nucleus is heavily restricted, and any soluble cargo accessing it passes through the nuclear pore complex (NPC). The NPC is a 125 MDa cylinder comprising multiple copies of approximately 30 nucleoporins (Nups), which span the whole nuclear envelope [159,160]. Nups enriched in hydrophobic FG repeats line the NPC central pore, forming a hydrogel plug only permeable to soluble proteins lighter than 40–60 kDa [159,161]. Any soluble proteins heavier than this require a nuclear transport receptor to pass through the NPC [162]; for vRNPs, this receptor is importin β1 [14,25,163,164].

Importin β1 cooperates with importin α and Ran GTPase to facilitate nuclear import of vRNPs [165]. The first step is the binding of importin α to the unconventional NLS on NP, which is exposed after the M1 dissociation [20]. Fluorescent tracking suggests that all eight vRNPs are imported as one bundle, which separates post-import [25]. Binding to the NLS releases the importin β-binding (IBB) domain of importin α, thus recruiting importin β to the complex [159]. Importin β1 interacts with the FG repeats lining the NPC central pore, allowing it (and by extension importin α and the vRNPs) to slide through the NPC and enter the nucleus [162]. The nascently imported importin β1 binds GTP-bound Ran (RanGTP), dissociating it and thus releasing the vRNP-importin α complex into the nucleus for viral transcription and replication [16,166]; whether importin α remains associated with vRNPs post-import is unknown [20].

## 5. Viral mRNA Transcription and Translation of Viral Cytosolic Proteins

**Figure 18:**
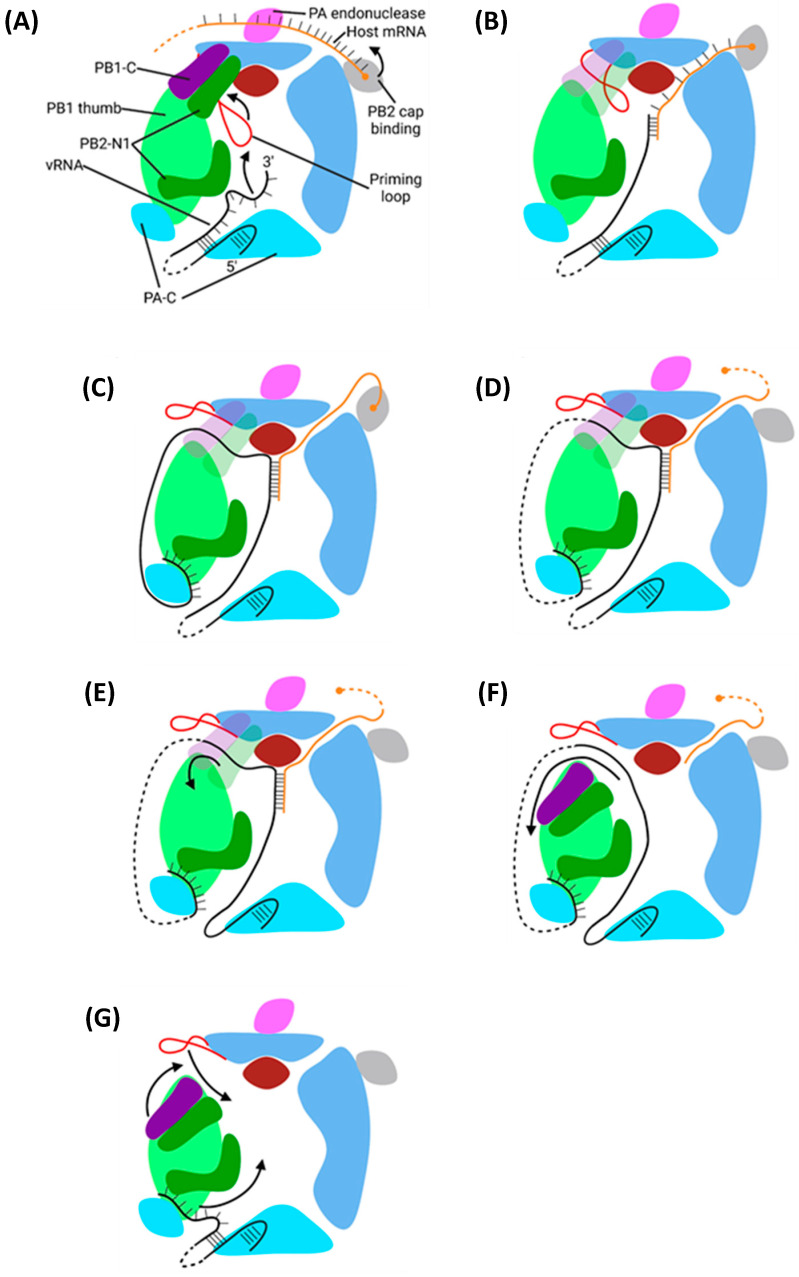
Overall model of viral mRNA transcription. (**A**): Transcription of viral mRNA starts with the cap snatching of host mRNA by the PB2 cap-binding domain (grey). The cap-binding domain is oriented such that mRNA 5′ nt 12–14 are cleaved by the PA endonuclease domain (pink) [167]. (**B**): The PB2 cap-binding domain rotates to position the nascently cleaved mRNA 3′ terminus into the active site, and stochastic retraction of the priming loop (red) through the template exit tunnel permits the formation of a five bp vRNA–mRNA duplex [48]. (**C**): Subsequent elongation forms a nine-bp duplex, after which the PB2 helical lid domain (maroon) forces the vRNA and mRNA strands down their respective exit tunnels [48]. The emerging vRNA binds a secondary binding site on the polymerase surface [46]. (**D**): The force generated by translocation dissociates the vRNA from the underlying oligomeric NP scaffold, which is progressively disassembled and then reassembled as the polymerase travels down the length of the vRNP [169]. Buckling of the viral mRNA dissociates it from the PB2 cap-binding domain [167]. (**E**): Transcription continues until the 5′ hook prevents further translocation of the template strand, and the viral mRNA is polyadenylated by reiterative stuttering [39]. (**F**): Mismatches in the A-U rich duplex are thought to separate the vRN–mRNA duplex, and the viral mRNA exits through the product exit tunnel. The PB1 C (purple) and PB2 N1 (dark green) domains rotate to open the template exit tunnel [39]. (**G**): The vRNA exits the tunnel in a skipping rope-like manner before the PB1 C and PB2 N1 domains pivot back to seal the tunnel [39]. With no template strand to provide steric hindrance, the priming loop re-enters the template exit tunnel [48]. The vRNA 3′ terminus dissociates from its secondary binding site and enters the active site cavity, returning the polymerase to its pre-initiation conformation [39,167]. Figure created with BioRender.com.

After nuclear import, the vRNP-resident viral polymerase transcribes viral mRNA from a vRNA template (Figure 18). In brief, nascently transcribed host m7G-capped mRNA binds PB2 and is cleaved by PA [167]. The cleaved mRNA enters the polymerase active site, base pairs with the vRNA 3′ terminus, and is elongated by two catalytic divalent metal cations coordinated by three conserved aspartate residues [48]. Force from translocation displaces the mRNA from PB2, and the emerging vRNA binds a secondary binding site on the polymerase surface [46]. Transcription continues uninterrupted until the tight binding of the vRNA 5′ hook to its binding pocket prevents further translocation, and the mRNA is polyadenylated by reiterative stuttering [39]. Eventually, the vRNA–mRNA duplex dissociates; the viral mRNA undergoes post-transcriptional modifications and nuclear export, and the vRNA exits the polymerase catalytic core in a skipping rope-like manner to return the polymerase to its original conformation for another round of transcription [39]. IAV polymerase adopts either a transcriptionally inactive or pre-initiation conformation. In the inactive conformation, the PB2 cap-binding domain is obstructed [47]; in the pre-initiation conformation, the cap-binding domain is accessible, and the PA endonuclease domain is correctly positioned to cleave the host mRNA [49]. The binding of the IAV polymerase to the vRNA promoter might switch the polymerase to an open conformation more permissible to the transcription [168].

### 5.1. Cap Snatching by PB2 and PA

Transcription of viral mRNA requires a primer derived from 5′ m7G capped host mRNA snatched from actively transcribing host RNA polymerase II (Pol II) [26,170]. The Pol II CTD comprises 52 YSPTSPS heptad repeats, which bind IAV polymerase through the PA CTD [171,172]. All heptad residues except the prolines are phosphorylated at different stages of host transcription; IAV polymerase binds the serine-5 phosphorylated (S5p) Pol II CTD, a hallmark of Pol II in transcription initiation when the m7G cap is readily accessible [172,173,174]. The PA CTD has two separate S5p binding sites. The first interacts with a single heptad, the second with residues from three continuous heptads; both sites establish numerous hydrogen bonds and hydrophobic interactions [171]. Exactly how IAV polymerase adopts a transcription- as opposed to replication-competent conformation remains unclear, but the binding to Pol II likely shifts the balance in favour of transcription [39,42].

The binding to m7G-capped host mRNA is performed by the PB2 cap-binding domain, which protrudes from and rotates about the polymerase body [167,168,175,176]. Proper functioning of the cap-binding domain requires the binding of the vRNA 5′ terminus to the polymerase, possibly by inducing an allosteric conformational change, which allows the aromatic and charged residues to stack with the m7G cap [177,178,179].

Once captured by the PB2 cap-binding domain, the host mRNA is cleaved by the PA endonuclease domain at a 5′-G^C-3′ site where G is nt 10–13; if no G is present at nt 10–13, the mRNA is instead cleaved at nt 12 [38,180,181]. As observed with the influenza B virus, after cleavage, the PB2 cap-binding domain rotates, and the mRNA 3′ terminus is forced into the polymerase active site through clashes with the PB2 lid domain [167]. Activation of the PA endonuclease domain also requires a conformational change induced by binding of the polymerase to the vRNA 5′ and 3′ termini [178].

### 5.2. Mechanism of Transcription by PB1

The IAV polymerase catalytic centre is in the PB1 active site cavity formed by the classical RdRp motifs pre-A (also called F), A, B, C, D, E, and F. At the centre of this cavity, two catalytic divalent metal cations (Mg^2+^ in vivo, Mn^2+^ in vitro) are coordinated by three aspartate residues [46]. Four tunnels extend from the central cavity: the template and NTP entrance tunnels and the template and product exit tunnels [47]. In the pre-initiation state, the PB1 priming loop is inserted into the active site through the template exit tunnel, and the vRNA 3′ terminus shifts between being in and out of the active site cavity [46,48].

#### 5.2.1. Initiation

Transcription initiation starts with the partial extrusion of the priming loop through the template exit tunnel via stochastic motion. This provides enough space in the active site cavity to accommodate the base pairing of the first 2 nt of the vRNA and cleaved host mRNA 3′ termini, which stabilises the duplex long enough to permit elongation [46,182].

#### 5.2.2. Elongation

**Figure 19:**
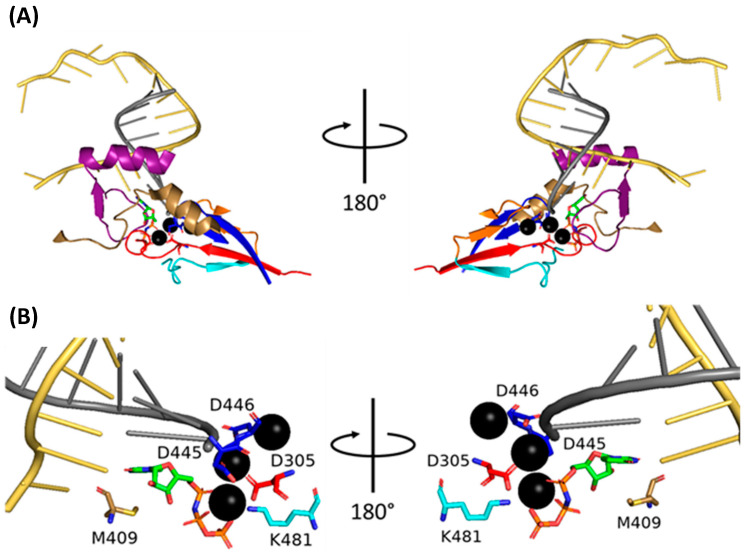
Ribbon structure of the A/little yellow-shouldered bat/Guatemala/060/2010 (H17N10) polymerase active site during elongation with key motifs highlighted. (**A**) vRNA in yellow; mRNA in grey; Mg^2+^ cations in black. Motif pre-A (residues 229–257, purple) binds the vRNA and channels free NTPs into the active site; motif A (296–314, red) contributes to the NTP entrance tunnel and coordinates the Mg^2+^ cations; motif B (401–422, sand) stabilises the base paring between the vRNA and incoming NTP; motif C (436–449, dark blue) also coordinates the Mg^2+^ cations; motif D (474–486, light blue) contributes to the NTP entrance tunnel; motif E (487–497, orange) probably stabilises the priming nucleotide [47]. (**B**) Close-up of the active site with catalytic residues is shown. The phosphates of the incoming NTP interact with two Mg^2+^ cations, positioned at the base of the active site by motif A D305 and motif C D445 + 446, with motif D K481 further stabilising this binding. M409 on motif D stacks behind the incoming NTP, stabilising the NTP-vRNA interaction [39]. While this structure shows three ions, the accepted mechanism of NTP addition involves two; given that the Mg^2+^ ions can move up to 5 Å [39], it is possible that the top and middle ion in the left-hand panel of (**B**) represent one highly mobile ion. Structure rendered in PyMol from PDB 6T0V [39].

NTP addition requires two Mg^2+^/Mn^2+^ cations coordinated by D305 of motif A and D445 and D446 of motif C (Figure 19) [39,183]. The current nucleotide addition cycle posits that the polymerase cycles between six states: 1. The active site is open and lacks free NTPs; 2. A free NTP base pairs with the vRNA; 3. The catalytic residues and Mg^2+^/Mn^2+^ cations close around the mRNA and NTP; 4. The NTP is incorporated into the mRNA; 5. Post-catalysis conformational changes open the active site; 6. The vRNA and mRNA strands are translocated forwards by one bp [184]. Translocation of the vRNA occurs via ratcheted Brownian motion, with a sharp turn in the RNA backbone preventing movement in the reverse direction [184].

vRNA translocation melts the promoter, causing extensive conformational changes in the promoter binding region and template entry tunnel, which widen the active site cavity, allowing for the growth of the vRNA–mRNA duplex [48]. Partial retraction of the priming loop via stochastic motion yields enough space in the internal cavity to permit the formation of a five-bp duplex. Translocation of the vRNA provides sufficient force for complete retraction, leaving enough space for a nine-bp duplex which is stabilised in the cavity by extensive van der Waals and polar interactions [48]. After nine bp clashes with Y207 on the PB2, the helical lid domain forces the vRNA and mRNA into their respective exit tunnels [48]. The addition of 11–15 nt exerts enough buckling force to release the mRNA 5′ terminus from the PB2 cap-binding domain, after which it emerges from the active site cavity into a basic channel, avoiding the PA endonuclease [167]. The emerging vRNA 3′ terminus is guided down a basic groove to a secondary binding site on the polymerase surface [39]. While the oligomeric NP scaffold can remain intact in the absence of vRNA, recent evidence suggests that the force from translocation progressively dissociates the scaffold as vRNA is pulled into the active site [169,185]. As the vRNA passes into, through, and out of the active site cavity, the oligomeric NP scaffold is incrementally disassembled and reassembled, causing the polymerase to travel down the length of the vRNP [169].

#### 5.2.3. Polyadenylation and Termination

**Figure 20:**
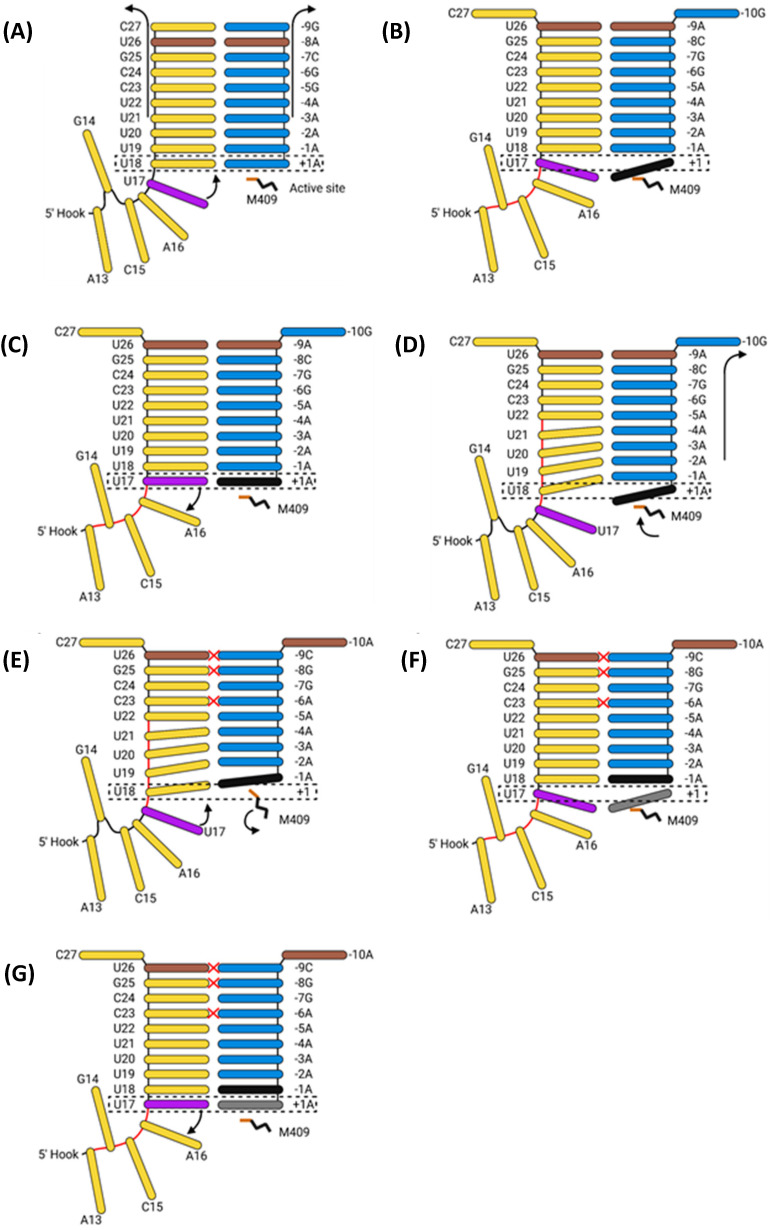
Polyadenylation of IAV mRNA. (**A**) Polyadenylation occurs by reiterative stuttering of an oligo(U) track at vRNA 5′ nt 17−22 [186]. U18 is the final vRNA nt to be transcribed without introducing tension on the linker (A13−U17), which connects the 5′ hook to the rest of the vRNA [39]. (**B**) After vRNA translocation, U17 (purple) enters the active site, introducing tension into the linker (regions under tension indicated in red). mRNA translocation frees the active site for another ATP (black) [39]. (**C**) The ATP is stabilised in the active site and incorporated into the mRNA with the aid of U17 and PB1 motif B M409 [39]. (**D**) The strain in the linker causes the vRNA to backtrack, returning the linker to its original conformation. Buttressing by M409 prevents mRNA backtracking, transferring the strain to the vRNA–mRNA duplex [39]. (**E**) The recently transferred strain and the rotation of M409 translocate the mRNA forward one base. This duplex rupturing and subsequent reformation introduces multiple mismatches (red crosses) [39]. (**F**) U17 re-enters the now unoccupied active site and is stabilised by another free ATP (grey) [39]. (**G**) The ATP is incorporated into the product strand shortly before U17 slips out of the active site, returning the system to the state in panel (**D**). Repetition of this process yields a polyadenylated viral mRNA [39]. Figure created with BioRender.com.

Polyadenylation of IAV mRNA occurs via reiterative stuttering at an oligo(U) track located at vRNA 5′ nt 17–22 (Figure 20) [186]. As previously mentioned, the 5′ vRNA terminus forms a 5′ hook that remains tightly bound to the polymerase [187]. When vRNA 5′ U17 enters the active site, it introduces strain into the A13-U17 [39]. An incoming ATP stabilises U17 in the active site long enough to permit its incorporation into the mRNA, after which the strain forces U17 out of the active site [39]. M409 on PB1 motif B prohibits the backtracking of the mRNA; the backtracking of the vRNA but not the mRNA strand transfers the strain to the duplex [39]. This strain and the rotation of M409 force the mRNA forward one base, rupturing and reforming the duplex, introducing multiple mismatches in the process [39]. U17 re-enters the now free active site, is stabilised by and incorporates another free ATP, and slips out of the active site. This cycle repeats until 30–180 adenosines have been incorporated, after which the vRNA–mRNA duplex dissociates [188]. The exact mechanism remains unknown, but it likely involves a conformational change in the polymerase to a state that cannot support the vRNA–mRNA duplex binding [39].

With the product strand now fully released, the vRNA promoter must return to its pre-initiation conformation to permit another round of transcription. This happens when the PB1 C and PB2 N1 domains rotate to open the template exit tunnel. The vRNA exits in a skipping rope-like manner before the PB1 C and PB2 N1 domains rotate back to seal the template exit tunnel [39]. The vRNA 3′ terminus then reforms the promoter duplex, and the priming loop re-enters the template exit tunnel, returning the polymerase to its pre-initiation conformation [39,48].

#### 5.2.4. Post-Transcriptional Processing and Translation of Soluble Viral Proteins

The mRNA from segments 7 and 8 are spliced to produce the mRNA for M2 and NEP, respectively [189,190,191]. Post-transcription, the m7G cap, poly(A) tail, and untranslated regions of the viral mRNA are associated with various RNA binding proteins, which recruit the mRNA to the NPC [192]. Here, the NXF1/NXT1, a heterodimer that binds both mRNA and the FG repeats lining the NPC, is recruited to the mRNA, and facilitates its passage through the NPC. Once in the cytosol, the mRNA is released for translation by cytosolic ribosomes [192].

Nascently exported viral mRNA is bound by poly(A) binding protein 1, which recruits the ribosome through a series of initiation factors [193]. Once the ribosome is assembled, the soluble viral proteins (PB2, PB1, PA, NP, M1, NEP) are translated directly into the cytosol and the transmembrane proteins (HA, NA, and M2) are trafficked to the ER [16]. The translation and trafficking of the viral transmembrane proteins is discussed later.

PB2 has a bipartite NLS comprising residues 448–496 and 736–739 [194]. PB1 and PA heterodimerise in the cytosol and are imported by Ran-binding protein 5, a member of the importin β superfamily [195,196,197]. The PB1 NLS comprises residues 187–211, and PA contains two NLSs between residues 124–139 and 186–247 [198,199]. Once imported, the PB1-PA heterodimer associates with PB2 to form the heterotrimeric viral polymerase [200]. NP contains three NLSs at residues 1–13, 198–216, and 327–345, which import NP via the classical nuclear import pathway [201,202,203,204]. The unconventional NLS at residues 1–13 is likely the primary signal for the import of free NP and vRNPs [57]. The M1 NLS comprises residues 101–105 and is recognised by importin α [205]. Owing to its small size, NEP diffuses through the NPC unaided [206].

## 6. Replication of vRNPs

For clarity, hereafter, ‘transcription’ refers to the synthesis of viral mRNA from vRNA, ‘primary replication’ refers to the synthesis of cRNA from vRNA, and ‘secondary replication’ refers to the synthesis of vRNA from cRNA.

### 6.1. Primary Replication

**Figure 21:**
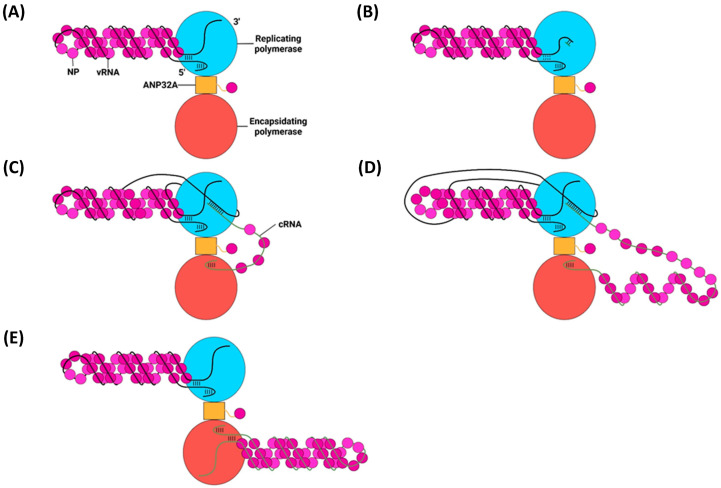
Primary replication at the level of vRNPs. (**A**) Primary replication requires a vRNP comprising a replicating polymerase (blue), vRNA (black), and NP (pink/purple) dimerised with an encapsidating polymerase (red) through host ANP32A (orange) [42]. The vRNA 3′ terminus is highly flexible and binds a secondary binding site on the replicating polymerase surface [39]. It is unknown if polymerase dimerisation occurs at a specific stage, although dimerisation likely encourages the formation of a replication-competent polymerase [208]. ANP32A also binds monomeric NP (purple/pink), ensuring NP is readily available for incorporation into the growing RNP [209]. (**B**) The vRNA 3′ terminus enters the active site, and the pppApG cRNA primer (green) is formed base-paired to nt 1-UC-2, stabilising the terminus in the active site and destabilising the promoter [210,211]. (**C**) Nucleotide addition occurs via the same mechanism of viral mRNA transcription [212]. A short segment of the vRNA template dissociates from the vRNP, possibly leaving behind the oligomeric NP scaffold [185]. While IAV polymerase moves down the length of the vRNP during viral mRNA transcription [169], it is unclear if this occurs during replication. After translocation through the active site, the emerging vRNA binds the pre-initiation secondary binding site and the NP scaffold [46]. The polymerase product exit tunnel is likely positioned such that the nascent cRNA is guided by a basic groove on ANP32A to the 5′ hook binding site in the encapsidating polymerase [213]. Any exposed cRNA is swiftly encapsidated after exit by NP tethered to ANP32A [209]. (**D**) As replication continues, the segment of dissociated vRNA moves along the vRNP in a zipper-like manner, travelling in a 3′ to 5′ direction [185]. The emerging cRNA continues to be encapsidated and starts to form the oligomeric NP scaffold of the cRNP [58]. (**E**) The segment of exposed vRNA eventually reaches the 5′ terminus. By an unknown mechanism, the vRNA 5′ hook dissociates from its binding pocket and enters the active site, after which it exits via the template exit tunnel to reassociate with its binding pocket [42]. After the 5′ hook passes through the active site, the cRNA 3′ terminus exits the replicating polymerase and binds the secondary binding site on the encapsidating polymerase, forming a complete cRNP [42]. Figure created with BioRender.com.

Replication of the negative-sense vRNA genome involves the generation of a positive-sense cRNA intermediate, which is then replicated into a progeny vRNA [27]. cRNA differs from viral mRNA in that it is an exact copy of vRNA, lacking an m7G cap and poly(A) tail, and is synthesised with different forms of initiation and termination [207]. Primary replication requires two IAV polymerases bridged by host ANP32A: one polymerase replicates the genome, and the other encapsidates the emerging cRNA to form a cRNP (Figure 21) [42]. IAV polymerase switches between transcription- or replication-competent conformations through an intermediate state with a blocked cap-binding domain and contracted core region [208]. Whether the intermediate state adopts the transcription or replication-competent conformation is presumed to rely on several molecular cues. Binding host Pol II may unblock the cap-binding domain and stabilise the transcription-competent conformation; binding ANP32A and free IAV polymerase may stabilise the replication-competent conformation [208].

**Figure 22:**
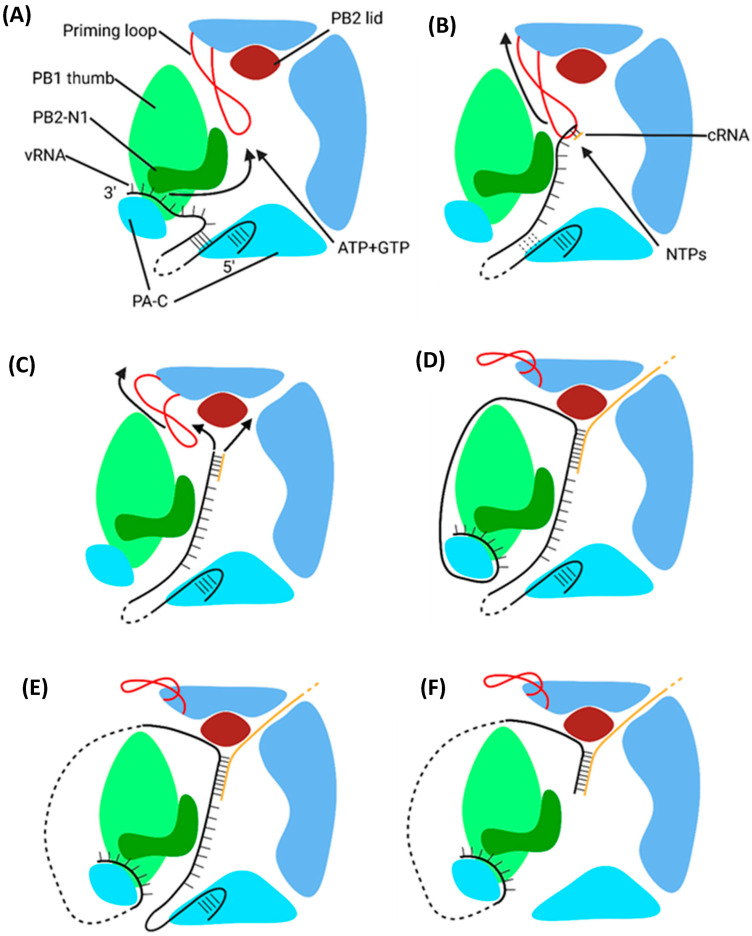
Primary replication at the level of the replicating polymerase. (**A**) vRNA primary replication starts with the replicating polymerase in the pre-initiation state, with the vRNA (black) 3′ terminus in the secondary binding site on the polymerase surface [46]. The priming loop (red) blocks the template exit tunnel with its tip positioned in the active site cavity [215]. (**B**) The 3′ terminus enters the active site, and the priming loop coordinates the formation of the pppApG cRNA primer (orange) base paired to vRNA 3′ nt 1-UC-2 [215]. The formation of the primer stabilises the vRNA in the active site and destabilises the vRNA promoter, encouraging replication initiation [210]. (**C**) Stochastic motion causes the priming loop to partially backtrack through the template exit tunnel, leaving enough space for a five bp vRNA–cRNA duplex in the active site [212]. (**D**) As elongation continues, the force from vRNA translocation fully retracts the priming loop from the template exit tunnel, which leaves enough space in the active site cavity for a nine-bp duplex. Steric clashes with the PB2 helical lid ensure that the vRNA and cRNA strands go down their respective exit tunnels [39]. The emerging cRNA forms a 5′ hook bound to the encapsidating polymerase (not shown) [213]. (**E**) With further elongation, the emerging vRNA binds the oligomeric NP scaffold it dissociated from, and the cRNA is encapsidated by NP, starting to form a cRNP (both omitted for clarity) [209]. (**F**) By an unknown mechanism, the vRNA 5′ terminus dissociates from its binding pocket and is replicated. The cRNA 3′ terminus exits the active site and binds the encapsidating polymerase, forming a complete cRNP. The vRNA 5′ terminus passes through the template exit tunnel and reforms the 5′ hook in the corresponding binding pocket, permitting another round of initiation [42]. Figure created with BioRender.com.

Primary replication utilises a de novo method of initiation, using the PB1 priming loop to form a pppApG primer base-paired to vRNA 3′ nt 1-UC-2 (Figure 22) [211,214]. The vRNA 3′ terminus either lies on the polymerase surface in the pre-initiation state or enters the active site to form the initiation state [210]. The formation of the pppApG primer by the priming loop stabilises the vRNA 3′ terminus in the active site and destabilises the 5′-3′ vRNA duplex, shifting the equilibrium to favour initiation [210,215]. Product strand elongation occurs via the same mechanism as that for transcription, with NTPs being incorporated by Mg^2+^/Mn^2+^ cations coordinated by D305, D445 and D446 [212]. The energy released by the base pairing of the vRNA–cRNA duplex breaks the vRNA promoter to continue elongation, and vRNA translocation forces the priming loop out of the template exit tunnel [48,182,216]. The product exit site of replicating influenza C polymerase is positioned such that the cRNA 5′ terminus is guided by a basic channel to its corresponding binding site on the encapsidating polymerase; although unconfirmed, this conformation is likely conserved with IAV polymerase [213]. While the vRNA 5′ hook must dissociate from its binding pocket to permit the replication of an exact copy of the vRNA, exactly how it achieves this remains to be elucidated [42].

#### cRNP Assembly

Despite being synthesised from the same template, cRNA is far less stable than viral mRNA in early-stage infection as it lacks the necessary viral proteins to protect it from degradation by host factors [60]. cRNA is stable when part of a cRNP, the formation of which requires an encapsidating polymerase and the co-transcriptional addition of NP via homo-oligomerisation [217,218]. Homo-oligomerisation of monomeric NP, recruited to the polymerase by ANP32A, is mediated by the insertion of the tail loop into the body domain of a neighbouring NP [58,209]. The tail loop is loosely attached to the NP body and adopts a wide array of conformations, permitting the NP–NP interaction to be maintained throughout the whole length of the cRNP [56,58]. A basic groove between the head and body domains of each NP monomer forms extensive sequence-independent electrostatic contacts with approximately 24 nt of the cRNA backbone [58,219]. Recruitment of cRNA to the basic groove is mediated by the 74–88 loop, which adopts an up or down conformation, only the latter of which permits RNA binding [220]. The down conformation partially opens the basic groove, and RNA binding (and possibly NP homo-oligomerisation) removes the C-terminal tail. Displacement of acidic residues in the C-terminal tail exposes the remaining basic residues in the groove, allowing for the rest of the cRNA to bind [220,221].

While monomeric NP homo-oligomerises as described above, it also readily forms homotrimers that are incapable of homo-oligomerisation and, thus, cannot be incorporated into the cRNP [222]. This issue is remedied by the phosphorylation of S165 and S407 by an unknown kinase, which creates a pool of monomeric NP by preventing the formation of important hydrogen bonds [223,224]. The phosphorylated NP is then sequestered by ANP32A, dephosphorylated by an unknown phosphatase to yield oligomerisation-competent monomeric NP, and incorporated into the growing cRNP before it can homotrimerise with other non-phosphorylated NP monomers [223].

### 6.2. Secondary Replication

**Figure 23:**
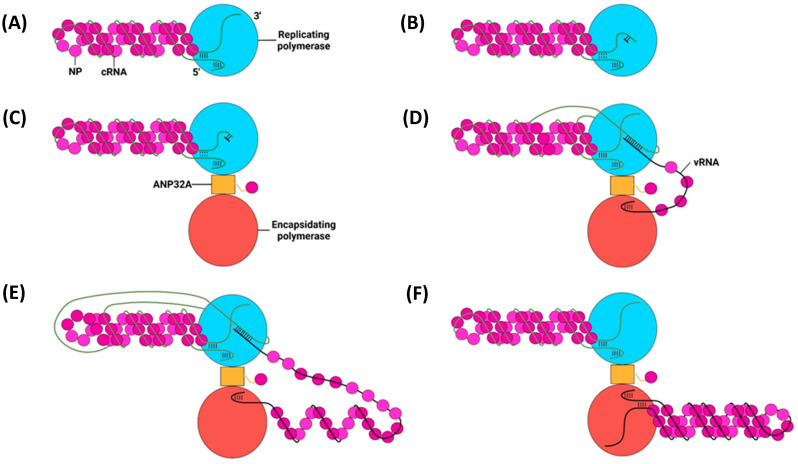
Secondary replication at the level of vRNPs. (**A**) Secondary replication starts with a free cRNP comprising a replicating polymerase (blue), cRNA (green) and NP (pink/purple). The cRNA 3′ terminus is highly flexible and binds a secondary binding site on the polymerase surface [39]. (**B**) The cRNA 3′ terminus enters the active site to initiate internal formation of the vRNA pppApG primer (black). Differences between the vRNA and cRNA 3′-5′ promoter duplexes positions the pppApG primer base paired to cRNA 3′ nt 4-UC-5 [226]. (**C**) Upon dimerisation with an encapsidating polymerase (red) via ANP32A (orange), the cRNA 3′ terminus backtracks relative to the pppApG primer, causing it to base pair with cRNA 3′ nt 1-UC-2 [228]. (**D**) Elongation causes a segment of cRNA to dissociate from the oligomeric NP scaffold and pass through the active site before rebinding its secondary binding site [229]. The exiting vRNA 5′ terminus is guided by ANP32A to its binding pocket in the encapsidating polymerase [213]. (**E**) The segment of dissociated cRNA moves along the cRNP scaffold in a zipper-like manner in a 3′ to 5′ direction, and the emerging vRNA is encapsidated to form a vRNP [58]. (**F**) By an unknown mechanism, the cRNA 5′ terminus dissociates from its binding pocket and is translocated through the active site before exiting via the template exit tunnel and rebinding its pocket [42]. The vRNA 3′ terminus emerges from the polymerase product exit tunnel and binds the secondary binding site on the surface of the encapsidating polymerase, forming a complete vRNP [42]. Figure created with BioRender.com.

**Figure 24:**
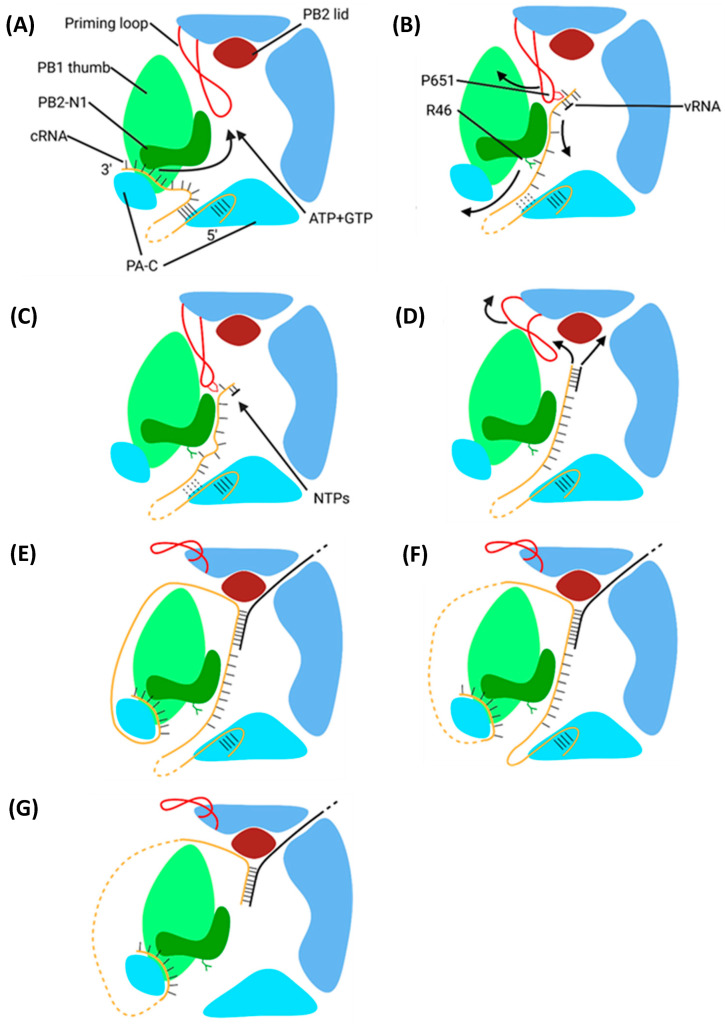
Secondary replication at the level of the replicating polymerase. (**A**) Secondary replication starts with the cRNA 3′ terminus bound to a secondary binding site on the polymerase surface. The cRNA promoter involves 3′ nt 12–14 in contrast to vRNA 3′ nt 10–12, leaving two extra unpaired nucleotides in the 3′ terminus [227]. (**B**) The cRNA 3′ terminus enters the polymerase active site cavity, and the pppApG primer base pairs to cRNA 3′ nt 4-UC-5 [215]. The cRNA 3′ terminus is kept in this stretched conformation by priming loop P651 and PB2-N1 R46 [228]. (**C**) Dimerisation with another polymerase via ANP32A causes a conformational change that shifts the PB1 thumb and PB2 N1 domains, moving P651 and R46, which backtracks the cRNA 3′ terminus [228]. The pppApG primer remains unmoved, however, and after the conformational change, it ends up base-paired to cRNA 3′ nt 1-UC-2 [228]. (**D**) Stochastic retraction of the priming loop permits a five-bp cRNA–vRNA duplex [212,216]. (**E**) The force of cRNA strand translocation fully extrudes the priming loop, permitting space for a nine-bp duplex. Beyond nine bp, the strands are forced apart by the PB2 helical lid domain [212]. The emerging cRNA 3′ terminus rebinds its secondary binding site on the polymerase surface while the emerging vRNA 5′ terminus forms a 5′ hook in the encapsidating polymerase (not shown) [213]. (**F**) As elongation continues, the emerging vRNA binds the oligomeric NP scaffold it dissociated from and the cRNA is encapsidated by NP, forming a cRNP (both omitted for clarity) [209]. (**G**) Eventually, the vRNA 5′ terminus dissociates from its binding pocket, enters the active site, is replicated, and then rebinds its pocket to return the replicating polymerase back to the conformation in panel A. The vRNA 3′ terminus exits the active site and binds the secondary binding site on the surface of the encapsidating polymerase, forming a complete vRNP [42]. Figure created with BioRender.com.

Secondary replication also requires a replicating and encapsidating polymerase bridged by ANP32A and utilises a de novo pppApG primer (Figure 23) [225]. Unlike primary replication, in secondary replication, the pppApG primer is formed internally, base-pairing to cRNA 3′ 4-UC-5 before being realigned to 1-UC-2 (Figure 24) [226]. Internal initiation occurs due to the alternative conformation of the cRNA promoter. Both the cRNA and vRNA promoter duplexes involve 5′ nt 11–13, but the cRNA duplex involves 3′ nt 12–14 as opposed to 3′ nt 10–12 of the vRNA duplex. This difference makes the cRNA 3′ terminus overshoot relative to the vRNA terminus, positioning cRNA 3′ nt 4-UC-5 in the active site [215,227]. This stretched conformation is maintained by P651 of the PB1 priming loop and R46 of the PB2 N1 domain [228]. Upon polymerase dimerisation, the priming loop and PB2 N1 domain undergo conformational changes, facilitating the backtracking of the cRNA 3′ terminus. The pppApG primer remains unmoved, as it is coordinated by D445 and D446, which do not undergo a conformational change; the pppApG primer is, thus, realigned to cRNA 3′ nt 1-UC-2 [228]. From here, the mechanism of vRNA elongation and vRNP formation is the same as that for primary replication. The energy from new base pairs between the cRNA and cRNA strands breaks the cRNA promoter, and the emerging vRNA is captured by the other polymerase and encapsidated [182,229].

### 6.3. The E627K Mutation

The PB2 E627K mutation is essential for the efficient replication of avian-adapted IAV in mammalian cells [230,231]. The function of this mutation revolves around the interaction between PB2 and ANP32A, which is essential for IAV polymerase dimerisation [209]. ANP32A comprises an N-terminal leucine-rich repeat region, a central domain, and a low-complexity acidic region (LCAR) [232]. The ANP32A LCAR interacts with the PB2 627 domain via many low-affinity hydrophobic and electrostatic interactions [233]. Avian-adapted PB2 contains E627, which disrupts a highly basic patch on the PB2 627 domain surface, thereby reducing the affinity between the ANP32A LCAR and PB2 627 domain [233]. In birds, this is compensated for by an avian-specific additional 33 residues in the LCAR, which provide a much larger interaction surface; thus, avian ANP32A is still able to efficiently interact with the avian-adapted E627-containing PB2 [233]. Because human ANP32A lacks these additional residues, there is no means to compensate for the disruptive effect of E627; the ANP32A-PB2 interaction, therefore, remains unstable and avian-adapted viruses cannot replicate efficiently in human cells. The E627K mutation in mammalian-adapted PB2 restores the highly basic patch on the PB2 627 surface, enabling a much stronger interaction between ANP32A and the mammalian-adapted PB2 [233].

## 7. Nuclear Export and Intracellular Trafficking of Progeny vRNPs

### 7.1. Nuclear Export

**Figure 25:**
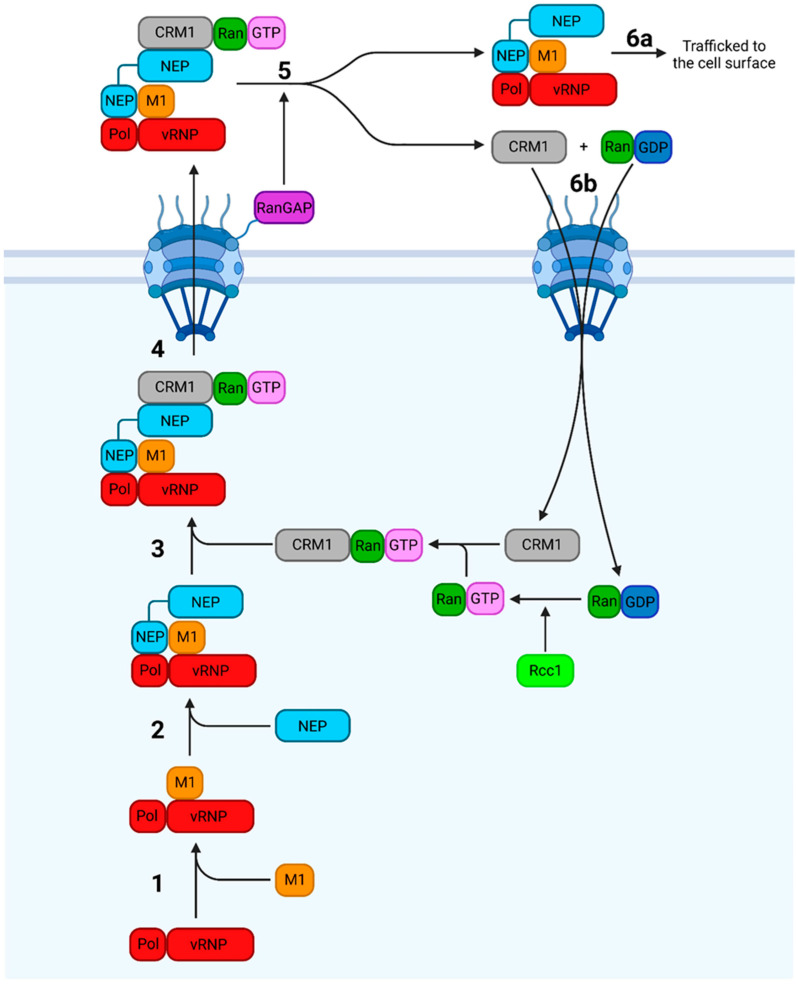
Nuclear export of progeny vRNPs. **1:** Nuclear export of vRNPs (red) occurs via the CRM1 pathway and starts with the binding of M1 (orange) to the vRNP [30]. **2:** The NEP (light blue) CTD binds to the polymerase (Pol) and M1, burying the M1 NLS to prevent nuclear re-import [30,70]. **3:** The NEP NTD binds CRM1 (grey), thus linking the progeny vRNP to the export receptor [234]. Binding of NEP to CRM1 only occurs if CRM1 is bound to RanGTP (dark green and pink); Ran is kept in the GTP-bound state by the RanGEF Rcc1 (light green), which is restricted to the nucleus [235]. **4:** CRM1-RanGTP guides the complex through the NPC into the cytosol, where **5:** RanGAP (purple) promotes the formation of RanGDP (dark green and dark blue), rendering CRM1 incapable of binding the vRNP–M1–NEP complex [235]. **6a:** The vRNP–M1–NEP complex is trafficked to the cell surface [234], and **6b:** CRM1 and RanGDP are re-imported to be used for another round of export [20]. It remains unclear if NEP remains associated with the vRNP post-export. Figure created with BioRender.com.

Progeny vRNPs are exported from the nucleus by CRM1, a nuclear export receptor that binds cargo with a nuclear export signal (NES) (Figure 25) [30]. NES recognition requires CRM1 to be bound to RanGTP, which mediates the transport of CRM1-bound cargo across the NPC [20]. Post-export, the RanGAP tethered to the cytosolic face of the NPC induces the formation of RanGDP, dissociating CRM1 from its cargo, which releases it into the cytosol. CRM1 and RanGDP are then imported back into the nucleus, where RanGDP is converted to RanGTP by the chromatin-bound RanGEF Rcc1 [20].

For progeny vRNPs, the cargo exported by CRM1 comprises a vRNP, M1, and NEP. Exactly how these components are organised has been debated, but the current model posits that NEP bridges the vRNP–M1 and CRM1–RanGTP complexes [70,234,236]. The vRNP-M1 interaction requires the NEP CTD, which possesses a cluster of glutamate residues with a protruding W78 that interacts with and buries the M1 NLS, preventing the immediate re-import of the complex after export [14,70,234,237]. The NEP CTD also recognises the vRNP- but not cRNP-resident polymerase, preventing the unnecessary export of cRNPs. This is possibly due to the different conformations the polymerase adopts upon vRNA versus cRNA binding [234,238]. NEP, now bound to the vRNP–M1 complex, is recognised by CRM1 via two NESs at residues 12–21 and 31–40 [30,74].

Progeny vRNPs associate with densely packed chromatin, bringing them into proximity with Rcc1; this targeting allows the progeny vRNPs to preferentially access host nuclear export machinery, thus enhancing replication efficiency [235,239]. Given its role in nuclear export, NEP would ideally only be expressed in late-stage infection when enough vRNPs have been replicated to make their export reasonable. NEP mRNA is produced by the inefficient splicing of NS1 mRNA, which acts as a molecular clock to coordinate the slow accumulation of NEP with that of progeny vRNPs during late-stage infection [240].

### 7.2. Intracellular Trafficking

For IAV virions to bud from the plasma membrane, the vRNPs must reach the cell surface. Both the distance and vRNPs are too large for this to occur by passive diffusion, so a model invoking active transport is required [29,241]. The exact mechanisms of post-export vRNP trafficking remain contested, with one of the two competing models proposing the usurping of the recycling endosome (RE) system [29] and the other the formation of a modified ER with liquid viral inclusion (LVIs) [28,242]; both models involve interactions with Rab11, which associates with exported vRNPs during all stages of cytoplasmic transport [243].

Rab11 is a host GTPase which directs the trafficking of REs (vesicles that return endocytosed material to the cell surface) along actin and microtubule networks through interactions with molecular motors [244,245,246]. In infected cells, Rab11 interacts with vRNPs (but not cRNPs) through the PB2 627 domain; the binding of Rab11 to vRNPs but not cRNPs may be another mechanism to ensure only vRNPs are incorporated into progeny virions [247].

#### 7.2.1. The Recycling Endosome Model

**Figure 26:**
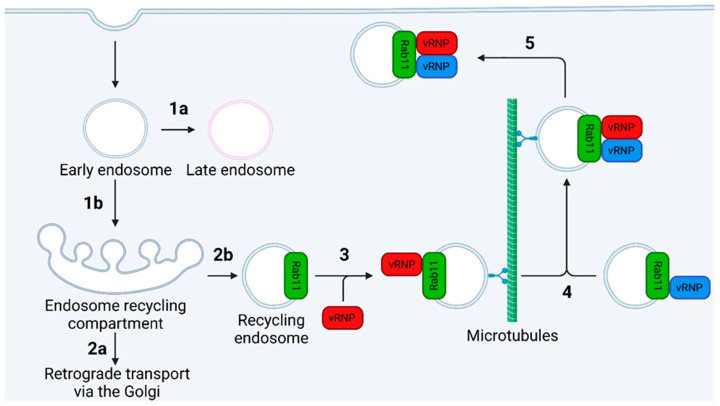
The recycling endosome model of vRNP trafficking. After endocytosis, early endosomes can either **1a:** mature into late endosomes or **1b:** fuse with the endosome recycling compartment (ERC) [245]. Those that fuse with the ERC either **2a:** undergo retrograde transport through the Golgi or **2b:** return to the plasma membrane as recycling endosomes (REs) [245]. **3:** Nascently exported vRNPs interact with Rab11, the defining protein of REs [29,246]. **4:** The vRNP-loaded REs are transported along microtubule tracks towards the plasma membrane [248]. While en route, the vRNP-Rab11 interaction sequesters vRNP-loaded vesicles into designated genome assembly compartment, forming an RE loaded with all eight IAV genome segments (only two are shown here for clarity) [250]. **5:** The REs with fully assembled genomes are then unloaded by an unknown mechanism from the microtubules to beneath the plasma membrane, the location of progeny IAV virion budding [16]. Figure created with BioRender.com.

The original RE model posited that vRNPs are loaded onto REs via a direct PB2-Rab11 interaction and trafficked to the cell surface along microtubule tracks (Figure 26) [29,248]. While infection with IAV alters transport along microtubule tracts by reducing Rab11-dynein association, the exact mechanism behind this remains incompletely understood [249]. There also exists a microtubule-independent mode of vRNP transport which may account for up to half of vRNP trafficking; again, the precise details behind this are poorly defined [249].

#### 7.2.2. The Modified ER Model

**Figure 27:**
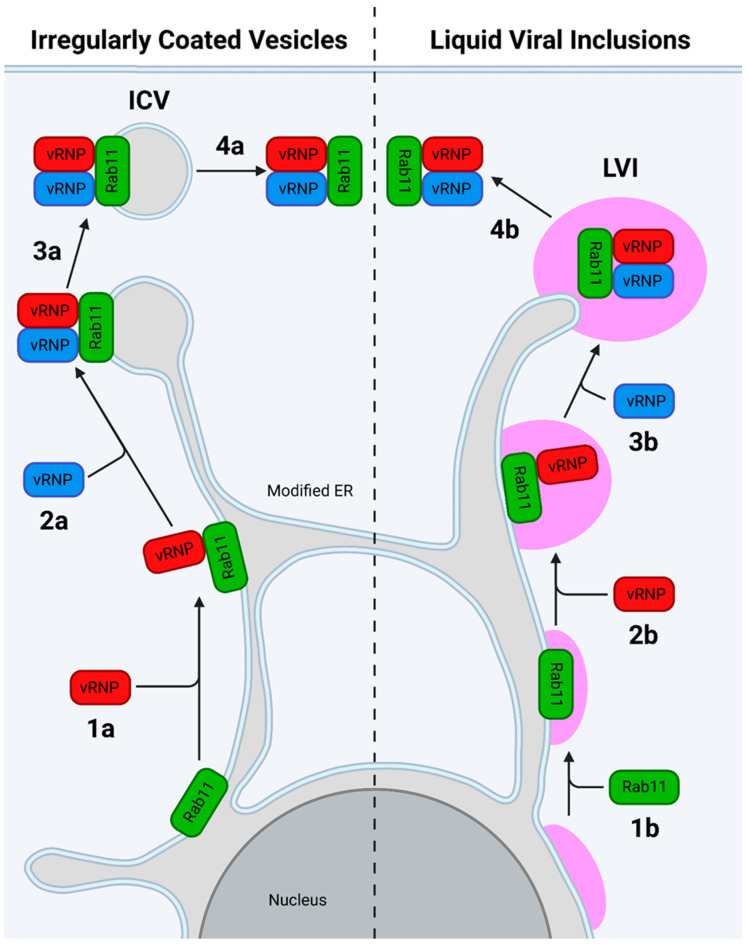
The modified ER model of vRNP trafficking. This model proposes that the Rab11–vRNP interaction forms a highly tubular ER, which permits trafficking of the viral genome beneath the plasma membrane by either irregularly coated vesicles (ICVs, left) or liquid viral inclusions (LVIs, right). **ICV: 1a:** The ICV model starts with the recruitment of Rab11 (green) and vRNP (red) to the modified ER membrane. **2a:** Additional vRNPs (blue) are recruited until a fully assembled genome bundle is formed (only two vRNPs shown for clarity) [242]. **3a:** The Rab11–vRNP complex buds from the ER to form ICVs, which **4a:** transport the fully assembled genome to the plasma membrane through Rab11 inactivation and interactions of the vRNPs with other viral proteins at the plasma membrane [242]. **LVI:** The LVI model involves liquid organelles (pink), regions adjacent to the plasma membrane that are distinct from the cytosol but lack a delimiting membrane. **1b:** Rab11 and **2b:** several vRNPs are recruited to these liquid organelles to form LVIs, concentrating the vRNPs into specific sites, thus promoting the formation of fully assembled genomes. The exchange of material between the LVI and cytosol is highly dynamic and does not follow any strict order [28]. **3b:** The LVIs localise to ER exit sites, which, due to the modified nature of the ER, are positioned beneath the plasma membrane. **4b:** The liquid property of the LVIs allows for the easy transfer of the fully assembled genome beneath the plasma membrane, and the contents of the LVIs are maintained by a constant influx and efflux of vRNPs and assembled genomes [28]. Recent evidence demonstrates that the LVIs may form from vRNP-loaded REs dissociated from microtubules by ATG9A [32]. Figure created with BioRender.com.

The newer model proposes that IAV infection redirects and impairs normal Rab11 function to induce the formation of a modified, tubular ER, which extends throughout the cell (Figure 27) [242]. In the original model, recently exported vRNPs bind Rab11 on the modified ER, forming irregularly coated vesicles that bud from the ER beneath the cell surface and transfer the vRNPs to the plasma membrane [242]. An updated model includes the presence of liquid viral inclusions (LVIs), regions adjacent to ER exit sites that lack a delimiting membrane yet remain separate from the cytosol. These LVIs provide a region to concentrate inter-segment RNA–RNA interactions and are maintained by a constant influx of vRNPs and efflux of fully assembled IAV genomes [28]. Recently, evidence has been found for a model in which host ATG9A promotes the dissociation of vRNP-loaded REs from microtubules. The REs then cluster at ER exit sites to form LVIs, which move along the length of the modified ER and fuse with each other en route [32].

## 8. Assembly of the IAV Genome

An infectious IAV virion contains eight unique vRNPs packaged perpendicular to the budding tip in a 7 + 1 conformation [251,252,253,254]. The vRNPs are exported as sub-bundles and then form a fully assembled genome while being trafficked to the plasma membrane [31]. The data currently support the compartmentalised model of genome assembly whereby the Rab11-vRNP interaction sequesters the vRNPs into a designated genome assembly compartment, with LVIs being a promising candidate [28,250].

**Figure 28:**
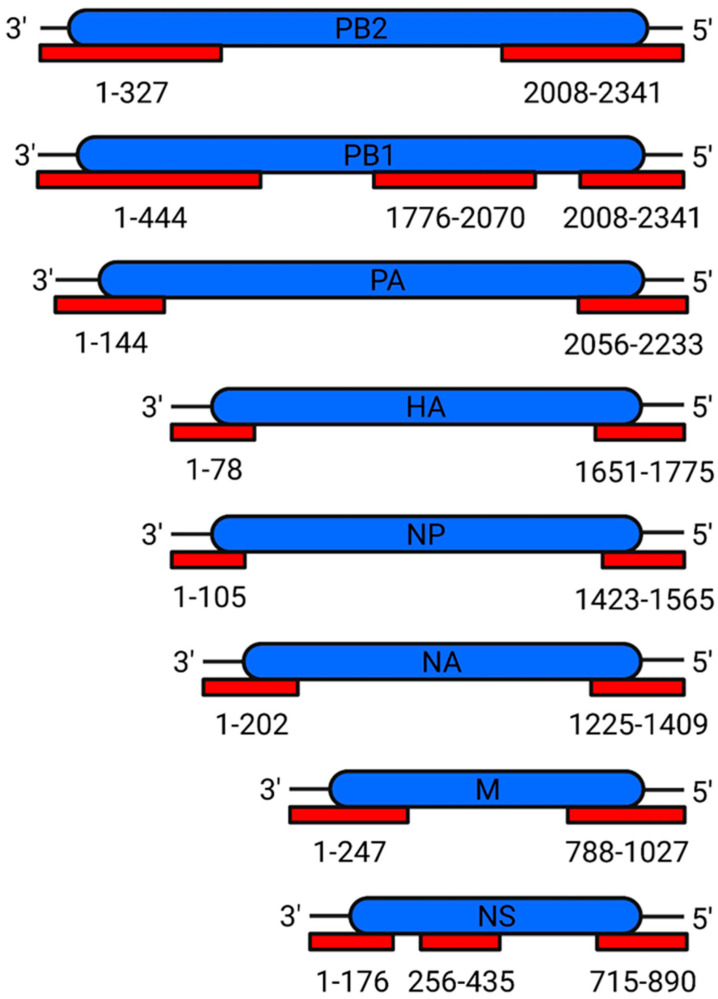
The IAV genome packaging signals. Each genome segment is displayed in the anti-sense orientation. Untranslated regions are in black; coding regions in blue; packaging signals in red. The packaging signals depicted are the maximum range from multiple strains, as reported by [258,259,260,261,262,263,264,265,266,267,268,269,270,271,272]. Figure created with BioRender.com.

IAV genome assembly is highly regulated, with the 7 + 1 pattern being so important that virions from IAV engineered to have seven segments still display the 7 + 1 pattern, incorporating host rRNA as the eighth segment [255]. This extreme degree of organisation led to the selective packaging model, which proposed the existence of selective packaging signals [256]. The first supporting evidence came in the form of defective interfering RNAs (diRNAs), which derive from vRNA and have large internal deletions but intact termini; diRNAs compete with full-length vRNA for genome packaging, implying that the packaging signal lies at the vRNA termini [257]. This model has since been confirmed with the identification of packaging signals in all eight genome segments (Figure 28).

### Mechanism of Packaging Signals

Intensive efforts notwithstanding, the exact mechanism of genome assembly remains incompletely understood [273]. The current model posits the existence of two types of signals: packaging signals, which are in non-coding regions and recruit individual vRNPs into the virion; and bundling signals, which reside in the terminal coding regions and coalesce vRNPs into one fully assembled genome [264]. Both signals utilise nucleotides from the 3′ and 5′ vRNA termini and are thought to adopt specific secondary structures, which form inter-segment base pairs, although direct evidence for this is currently lacking [263,264,274,275]. A distinct set of NP residues is thought to facilitate the adoption of the vRNA secondary structure necessary for inter-segment interactions [276]. These interactions form an extensive and redundant network capable of resisting small changes to the nucleotide sequence [274,277,278,279].

## 9. Translation and Trafficking of IAV Membrane Proteins

### 9.1. Translation and Folding

The IAV transmembrane proteins (HA, NA and M2) are unstable in the cytosol and are instead translated into the ER with their ectodomains undergoing co-translational glycosylation and their TMDs being transferred to the ER membrane. In short, ribosomes translating proteins with an ER signal sequence are targeted to the ER-resident translocon by signal recognition particle (SRP) [280,281]. The signal peptide is then transferred to the ER membrane, cleaved for HA, and the remainder of the protein is translated into the ER lumen [50,282]. The emerging polypeptide is co-translationally glycosylated by oligosaccharyltransferase (OST), and repeated cycles of calnexin (CNX) and calreticulin (CRT) binding aid proper protein folding [283,284]. After the TMD has been translated into the translocon, it is transferred to the ER membrane, from which it is trafficked to the Golgi for further processing [282,285].

#### 9.1.1. HA

HA has an ER signal sequence at the N-terminal 15–19 residues (the exact length varies according to subtype) [54]. This sequence is cleaved, and the remainder of the protein is translated into the ER lumen until the C-terminal TMD enters the translocon. The TMD is then transferred to the ER membrane, leaving HA with an ER-lumen resident ectodomain and a short cytoplasmic tail [41,50]. HA monomers fold from the top down, forming six folding intermediates [24,285,286,287]. At least five glycans are required to prevent the formation of protein aggregates, which are unable to leave the ER [288]. HA trimers form from a pool of pre-translated monomers in the ER approximately 7–10 min post-translation [52,289]. The trimers are anchored to the membrane by a tri-helical bundle, which is connected to the ectodomain by a short linker [51]. The helices of this bundle rotate, allowing the ectodomain to tilt over 50° relative to the central axis of the trimer; this flexibility may aid the pH-dependent dilation of HA1 required for viral–endosomal membrane fusion [51].

#### 9.1.2. NA

NA has an N-terminal hydrophobic domain, which functions as both an ER signal sequence and TMD [290]. This sequence is flipped before being inserted into the ER membrane, leaving fully translated NA with a short cytoplasmic tail, TMD, and ER lumen-resident ectodomain [41]. The lack of a definitive NA stalk and TMD structure prohibits detailed analysis on NA folding. Nonetheless, NA requires at least three glycans to interact with CNX and CRT co- and post-translationally to reverse the formation of aberrant disulphide bonds and protein aggregates [291]. NA tetramerisation may result from the pairing of two NA dimers via interactions involving the TMD [291].

#### 9.1.3. M2

M2 lacks an N-terminal signal sequence, instead utilising its TMD to leave M2 with an N-terminal ER lumen-resident tail and a C-terminal cytoplasmic tail [41,292]. The proper functioning of M2 requires the formation of a tetrameric transmembrane helical bundle [293,294,295]. Exactly how M2 homotetramerises, remains to be elucidated, but it likely involves inter-helical hydrogen bonds and cation-π interactions, which drive monomer-to-dimer and dimer-to-tetramer formation [293,296].

### 9.2. Trafficking to the Plasma Membrane

Proteins destined for the Golgi are first transported to the ER-Golgi intermediate compartment (ERGIC) in COP-II coated vesicles that bud from ER exit sites and are trafficked to the ERGIC in a GTP-dependent manner [297,298,299]. Transport from the ERGIC to the Golgi is of a much longer range and occurs along microtubule tracks [300,301].

HA is proteolytically cleaved at the Golgi into HA1 and HA2 in a step necessary for IAV infectivity [302]. The cleavage site protrudes from the HA surface and is either monobasic or polybasic [303,304]. Polybasic sites emerge from monobasic sites via nucleotide insertions reliant on polyadenine tracts and stem-loop structures in the region encoding the cleavage site [305]. Such features are found across multiple HA subtypes, but only in H5 and H7 are they found in the region encoding the cleavage site, explaining why strains with polybasic cleavage sites are currently restricted to H5 and H7 subtypes [305]. Cleavage of the monobasic site occurs at R^G, whereas cleavage of a polybasic site occurs at R-X-K/R-R^G [303,304]. Monobasic sites are cleaved by human airway trypsin-like (HAT) protease and transmembrane protease, serine 2 (TMPRSS2); in humans, both HAT and TMPRSS2 expression is limited to the respiratory tract, meaning that infection with IAV containing monobasic HA cannot spread outside of the respiratory tract [306,307]. Polybasic HA is cleaved by furin, a protease expressed ubiquitously throughout the body; this enables IAV expressing polybasic HA to be cleaved by tissues outside the respiratory tract and cause systemic infection [308,309].

HA, NA, and M2 all localise to the plasma membrane independently of other viral proteins [310,311,312]. The HA TMD sequesters HA into vesicles that bud from the trans-Golgi network [313]; NA possesses a similar sequence, which is extended over multiple regions of the TMD [314]. These vesicles reach the plasma membrane via a Rab cascade, whereby one compartment transits to another by recruiting the RabGEFs required for the next compartment [315]. HA and NA are, thus, transported through a series of different compartments until they reach Rab17- and Rab23-positive vesicles, which deliver them to the plasma membrane [34].

## 10. Budding of Progeny IAV Virions

**Figure 29:**
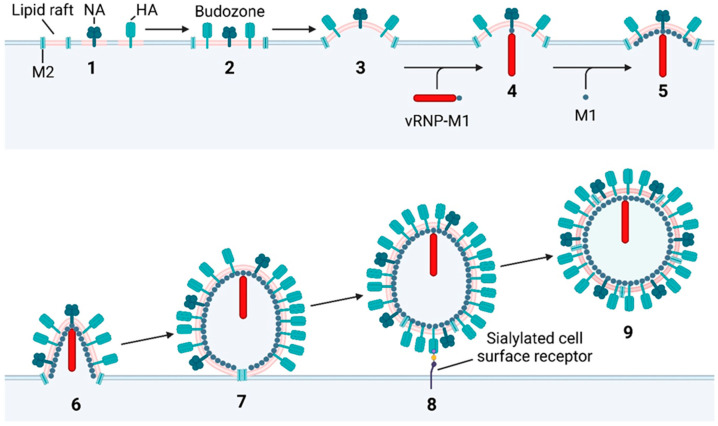
Overall model of IAV virion budding. **1**: Transmembrane viral proteins trafficked to the plasma membrane reside in (for HA and NA) or around (for M2) cholesterol-rich lipid rafts [43]. **2**: These lipid rafts coalesce into a budozone, with HA and NA distributed throughout and M2 lining the circumference [318,319]. **3**: HA and NA induce viral budding independently of other proteins [44]. **4**: The vRNP–M1 complex is recruited to the budozone through interactions with the M2 cytoplasmic tail [33]; the vRNP–M1 complex may be subsequently transferred to the HA and NA cytoplasmic tails, but this remains speculative [316]. While all eight genome segments are recruited as one bundle, only one segment is depicted for clarity. **5**: The HA cytoplasmic tail also seeds oligomerisation of soluble M1, which forms a helical matrix layer beneath the budding membrane [320]. **6**: The energy and structural support provided by this is assumed to allow the membrane to protrude further from the surface [157]. As it protrudes, the area of unincorporated budozone shrinks, and with it, the ring of M2 tetramers bound at its edge [35]. **7**: Once all of the budozone is incorporated into the nascent viral membrane, M2 switches from a high-cholesterol to low-cholesterol environment, inducing a conformational change which causes membrane curvature in the opposite direction of budding, resulting in membrane scission [33,35,69]. **8**: The viral and plasma membranes are now completely separated, but the nascent virion remains attached to the cell surface through HA-binding sialylated host cell surface receptors [36]. **9**: The sialidase activity of NA releases the sialic acid from the underlying receptor, freeing the virion from the infected cell [69,317]. Figure created with BioRender.com.

With all the required viral components present at the plasma membrane, progeny virions start to bud from the cell surface (Figure 29). To summarise, HA, NA, and M2 trafficked to the plasma membrane localise to lipid rafts which coalesce into a budozone [43]. HA and NA induce membrane curvature and recruit the viral genome through their cytoplasmic tails [33,316]. Cytoplasmic M1 forms helical oligomers, which is thought to provide the energy required to force the membrane outwards [157]. Once all the budozone is incorporated into the nascent viral membrane, M2 undergoes a conformational change, which induces membrane scission [35,69]. NA removes the terminal Neu5Ac residue from any glycans that HA may be bound to, freeing the virion into the extracellular matrix [36,317].

### 10.1. Association of IAV Transmembrane Proteins with Lipid Rafts

IAV virions bud from lipid rafts, cholesterol- and sphingolipid-enriched regions of the plasma membrane with important roles in apical trafficking in polarised cells [321,322,323].

HA-lipid raft association is mediated by the HA cytoplasmic tail and TMD [324,325]. After trimerisation, three conserved cysteine residues in the TMD and cytoplasmic tail are modified with palmitoyl, a highly hydrophobic saturated fatty acid [326,327]. The accumulation of HA in lipid rafts also activates the MAPK cascade; this may promote the nuclear export of vRNPs in an auto-regulatory mechanism, which induces vRNP export once all the necessary viral components are present at the budozone [328].

The NA TMD, which remains unmodified by fatty acids, is responsible for NA-lipid raft association [314].

Being a soluble protein, M1 is neither inserted into the membrane nor possesses a membrane-targeting sequence [329,330]; instead, electrostatic interactions between the M1 NTD and the HA, NA, and M2 cytoplasmic tails recruit M1 to the budozone [331,332]. The simultaneous interactions of the M1 CTD with vRNPs and the M1 NTD with viral membrane proteins recruit the fully assembled viral genome to the budozone [65,331,333].

M2, which is proposed to accumulate at the edge of lipid rafts and, thus, the budozone, binds to the cholesterol present in lipid rafts via the TMD base and an amphipathic helix in the cytoplasmic tail [12,35,318,334]. A cysteine residue in this helix is S-acylated, further stabilising the M2-lipid raft association [327]. Each M2 tetramer binds two cholesterol molecules in a proximal or diagonal conformation (binding to adjacent or opposing M2 monomers, respectively). The increased stability of the proximal conformation is thought to cause the clustering of M2 at the edge of the lipid rafts [334].

### 10.2. Induction of Budding

IAV protein expression causes the lipid rafts to coalesce into budozones, from which progeny virions bud [43]. Exactly how budding is initiated remains unclear, but both HA and NA intrinsically induce membrane curvature; co-expression with segment 7 (M1 and M2) is required to form and release virus-like particles [44]. Due to favouring different types of curvature, HA tends to localise to the shaft of the budding virion, whereas NA localises to the tip, the latter ideally positioned to aid virion release post-scission [44]. M1 is recruited to the budozone when cytoplasmic M1 binds the cytoplasmic tail of budozone-resident M2 [33]. M1 is then transferred to the HA and NA cytoplasmic tails, initiating M1 oligomerisation and virion budding [335].

### 10.3. Packaging of vRNPs

While not essential for budding, vRNPs are required to produce infectious virions [331]. M1 recruits vRNPs to the budozone through interactions between its mid-domain and both vRNA and NP [336,337]. Once recruited to the budozone, interactions with the HA, NA, and M2 cytoplasmic tails are presumed to mediate the recruitment of vRNPs into the budding virion [316,325].

### 10.4. M1 Oligomerisation

The interior of IAV virions is coated with a helical array of oligomerised M1 [45,338,339,340]. Upon oligomerisation, the M1 CTD transitions to a state capable of forming hydrophobic interactions with the NTD of an adjacent M1 monomer, recruiting it to the growing oligomer [341]. This processive model of assembly buries a large surface area, which likely releases the energy required for the protrusion of the virion from the cell surface [157].

### 10.5. M2-Mediated Scission

M2 drives viral–plasma membrane scission by suddenly inducing curvature in the opposite direction to budding. This curvature is induced by the insertion of an amphipathic helix into the membrane, with the effect being stronger in low-cholesterol environments [35,69]. As mentioned above, M2 tetramers are proposed to form a ring around the budozone [334]. Budding draws the cholesterol-enriched budozone away from the plasma membrane, thereby decreasing the circumference of the putative M2 ring [35]. Once enough budozone is incorporated into the budding virion, the cholesterol concentration of the region immediately surrounding M2 drops below the required threshold. With the cholesterol concentration now low enough, the membrane curvature induced by the insertion of the M2 amphipathic helix overcomes the curvature in the opposite direction induced by budding; this sudden and concentrated change in curvature is sufficient to cause membrane scission, separating the viral and plasma membranes [35]. While the M2 amphipathic helix is inserted into the membrane in high-cholesterol environments, its effect is much weaker [342]. Thus, it is only after enough of the cholesterol-enriched budozone has been drawn into the budding virion and away from M2 that the effects of amphipathic helix insertion is enough to cause scission [35]. It remains unclear whether one or multiple progeny virions bud from the same budozone.

### 10.6. NA-Mediated Release

Immediately post-scission, IAV virions remain attached to the cell surface due to HA-binding sialic acid on glycosylated surface proteins. NA cleaves the terminal Neu5Ac from the underlying glycan, releasing the virion from the cell [36,62]. NA has additional roles outside of virion release, preventing virion aggregation by removing the Neu5Ac from glycosylated HA and NA [317,343]. It is also essential for the movement of virions through the mucin-rich respiratory epithelium; mucins are heavily glycosylated extracellular proteins, which act as decoys by binding IAV virions, thus reducing virion-cell binding [344].

## Data Availability

Not applicable.
